# HIV infects astrocytes in vivo and egresses from the brain to the periphery

**DOI:** 10.1371/journal.ppat.1008381

**Published:** 2020-06-11

**Authors:** Victoria Lutgen, Srinivas D. Narasipura, Hannah J. Barbian, Maureen Richards, Jennillee Wallace, Roshanak Razmpour, Tetyana Buzhdygan, Servio H. Ramirez, Lisa Prevedel, Eliseo A. Eugenin, Lena Al-Harthi

**Affiliations:** 1 Department of Microbial Pathogens and Immunity, Rush University Medical Center, Chicago, Illinois, United States of America; 2 Department of Pathology and Laboratory Medicine, The Lewis Katz School of Medicine at Temple University, Philadelphia, Pennsylvania, United States of America; 3 Department of Neuroscience, Cell Biology and Anatomy, The University of Texas Medical Branch, Galveston, Texas, United States of America; Emory University, UNITED STATES

## Abstract

HIV invades the brain during acute infection. Yet, it is unknown whether long-lived infected brain cells release productive virus that can egress from the brain to re-seed peripheral organs. This understanding has significant implication for the brain as a reservoir for HIV and most importantly HIV interplay between the brain and peripheral organs. Given the sheer number of astrocytes in the human brain and their controversial role in HIV infection, we evaluated their infection in vivo and whether HIV infected astrocytes can support HIV egress to peripheral organs. We developed two novel models of chimeric human astrocyte/human peripheral blood mononuclear cells: NOD/*scid*-IL-2Rgc *null* (NSG) mice (huAstro/HuPBMCs) whereby we transplanted HIV (non-pseudotyped or VSVg-pseudotyped) infected or uninfected primary human fetal astrocytes (NHAs) or an astrocytoma cell line (U138MG) into the brain of neonate or adult NSG mice and reconstituted the animals with human peripheral blood mononuclear cells (PBMCs). We also transplanted uninfected astrocytes into the brain of NSG mice and reconstituted with infected PBMCs to mimic a biological infection course. As expected, the xenotransplanted astrocytes did not escape/migrate out of the brain and the blood brain barrier (BBB) was intact in this model. We demonstrate that astrocytes support HIV infection in vivo and egress to peripheral organs, at least in part, through trafficking of infected CD4+ T cells out of the brain. Astrocyte-derived HIV egress persists, albeit at low levels, under combination antiretroviral therapy (cART). Egressed HIV evolved with a pattern and rate typical of acute peripheral infection. Lastly, analysis of human cortical or hippocampal brain regions of donors under cART revealed that astrocytes harbor between 0.4–5.2% integrated HIV gag DNA and 2–7% are HIV gag mRNA positive. These studies establish a paradigm shift in the dynamic interaction between the brain and peripheral organs which can inform eradication of HIV reservoirs.

## Author summary

HIV latency and residual low-level HIV replication is a major obstacle towards an HIV cure. HIV infects the brain in acute disease yet it is unknown whether long lived-infected brain cells release productive virus that can egress from the brain to re-seed peripheral organs and whether astrocytes are productively infected in vivo. We demonstrate astrocyte-initiated HIV spread from the brain to the spleen and lymph nodes, likely through T cell trafficking out of CNS and into peripheral organs. Additionally, brain sections from patients on cART show HIV integration in astrocytes. Collectively, given that astrocytes constitute ~60% of brain cells and even with a conservative rate of infection at >3%, astrocytes can be a significant reservoir for HIV. As such, cure initiatives must consider the contribution of the CNS to ongoing HIV replication within and outside of the brain.

## Introduction

HIV latency and/or residual low-level HIV replication is a major obstacle towards an HIV cure. Combined anti-retroviral therapy (cART) intensification alone has not been able to eliminate the latent reservoir pool [1–3]. Much attention is focused on the role of CD4^+^ resting T cells in HIV latency [4,5]. However, other cellular reservoirs (e.g. sources of inducible HIV) capable of disseminating HIV virus remain including the CNS [6–8]. HIV invades the brain within approximately two weeks of infection, as demonstrated by both animal [9] and human [10] studies. The consequence of this neuroinvasion is the induction of inflammatory responses that culminate in the manifestation of HIV-Associated Neurocognitive Disorders (HAND), persistence of HIV in the CNS at a steady state despite combination antiretroviral therapy (cART), and HIV compartmentalization as indicated by the presence of HIV genetic sequences in the CNS that are distinct from those in the plasma and lymphoid tissue; although the exact source of HIV remains unidentified, some of these virus are macrophage-tropic [11–15]. The brain is not an immune privileged site with evidence demonstrating presence of a CNS lymphatic system with neuroimmune interactions occurring even under normal physiological conditions [16,17]. HIV neuroinvasion likely occurs through trafficking of HIV infected immune cells into the CNS and dissemination of HIV into the brain, where it then can infect resident brain cells (microglia, macrophages and to a lesser extent astrocytes) [18,19]. It is unknown whether long lived-infected brain cells (resident immune cells, microglia, macrophages and astrocytes) release productive virus that can egress from the brain to re-seed peripheral organs. This understanding has significant implications for HIV cure initiatives as egress of HIV from the brain to periphery may be another significant hurdle towards a cure and may also inform the source of HIV viral blips.

Although a number of animal models exist to address HIV in vivo [20], none can be used to assess whether HIV in the brain can egress to the periphery. We developed a chimeric human astrocyte/human PBMC mouse model to assess the role of astrocytes in supporting productive HIV replication in vivo and their role in disseminating HIV to tissues outside of the brain. We focused on astrocytes because astrocytes are the most abundant cell type in the brain and are pivotal to brain homeostasis. Although astrocytes do not support classical HIV entry through CD4/gp120 fusion, primarily because they are CD4 negative [21], they support HIV entry via cell-to-cell contact with infected CD4+ T cells as been shown in vitro though this phenomenon remains to be found in vivo [22,23] and through receptor-mediated endocytosis [24,25]. Putative binding factors have been described, the mannose binding protein [26], a 65kD membrane protein [27] and a 260kD protein [28]. The majority of HIV virions are degraded within the endosome of astrocytes, however, events that disrupt endosomal maturation allow for low levels of viral escape from the endocytic compartment [24,26,29] and eventual integration into the host DNA. Indeed, agents that disrupt endosome to lysosome maturation such as chloroquine or bafilomycin enhance HIV entry into astrocytes [29,30]. Non-classical, low-efficiency entry of HIV into astrocytes allows for reverse transcription and integration of HIV. The rate of integrated HIV DNA into astrocytes, based on in vitro [31] and ex vivo (post-mortem tissue from HIV infected individuals [32,33]) studies is approximately 1–3%. Others have estimated the rate to be close to 20% in HIV infected brain when astrocytes are in close proximity to macrophages [34], however, this may be elevated as a result of potential contamination of pure population of astrocytes near macrophages.

We previously demonstrated that astrocytes support low level of HIV infection in vitro and are restricted in productive HIV replication due to robust expression of Wnt/β-catenin signaling. β-catenin restricts HIV transcription by forming a multiprotein complex on the HIV promoter at -143nt site to interfere with RNA polymerase II processing [35]. This multiprotein complex consists of β-catenin, its transcriptional partner TCF-4 and the nuclear matrix protein SMAR-1 which pull the HIV DNA away from the nuclear matrix and from RNA pol II, disrupting transcription [36]. Inflammatory signals (IFNγ, GM-CSF, methamphetamine) and HIV itself (Tat) inhibit β-catenin signaling in astrocytes potentially leading to an inflammatory or activated state of astrocytes and setting the stage for the ability of astrocytes to provide bursts of productive HIV [37–39]. HIV DNA does integrate in astrocytes and exhibits features of latency as defined by the reactivation of infectious provirus, detection of low level HIV transcripts without protein expression, and epigenetic regulation of HIV DNA, specifically class I histone deacetylases (HDACs) and a lysine-specific histone methyltransferase, SU(VAR)3-9, silence HIV transcription in astrocytes [31]. Collectively, these studies demonstrate that astrocytes are infected by HIV, albeit at a low rate (1–3% of cells).

Given the sheer number of astrocytes in the human brain, estimated in the billion to trillion range [40] and their controversial role in HIV infection, we evaluated their infection in vivo and whether HIV infected astrocytes can support HIV egress to peripheral organs. To this end, we developed two novel models of chimeric human astrocyte/human peripheral blood mononuclear cells: NOD/*scid*-IL-2Rgc *null* (NSG) mice (huAstro/HuPBMCs). Specifically, we transplanted HIV (non-pseudotyped or VSVg-pseudotyped) infected or uninfected primary human fetal astrocytes (NHAs) or an astrocytoma cell line (U138MG) into the brain of neonate or adult NSG mice and reconstituted the animals with human peripheral blood mononuclear cells (PBMCs). We also transplanted uninfected astrocytes into the brain of NSG mice and reconstituted with infected PBMCs to mimic a biological infection course. Our findings demonstrate that astrocytes support productive HIV infection in vivo, support HIV spread from the brain through immune cells trafficking to peripheral organs, and that HIV trafficking between the brain and the periphery is a dynamic two-way process (brain to peripheral organs and peripheral organs to brain) with significant implications for viral evolution and re-seeding of HIV in peripheral organs.

## Results

### HIV uninfected and HIV infected human astrocytes engrafted into NSG mice survive and do not migrate outside the brain and there is no evidence for blood brain barrier disruption in this model

**Fig 1:**
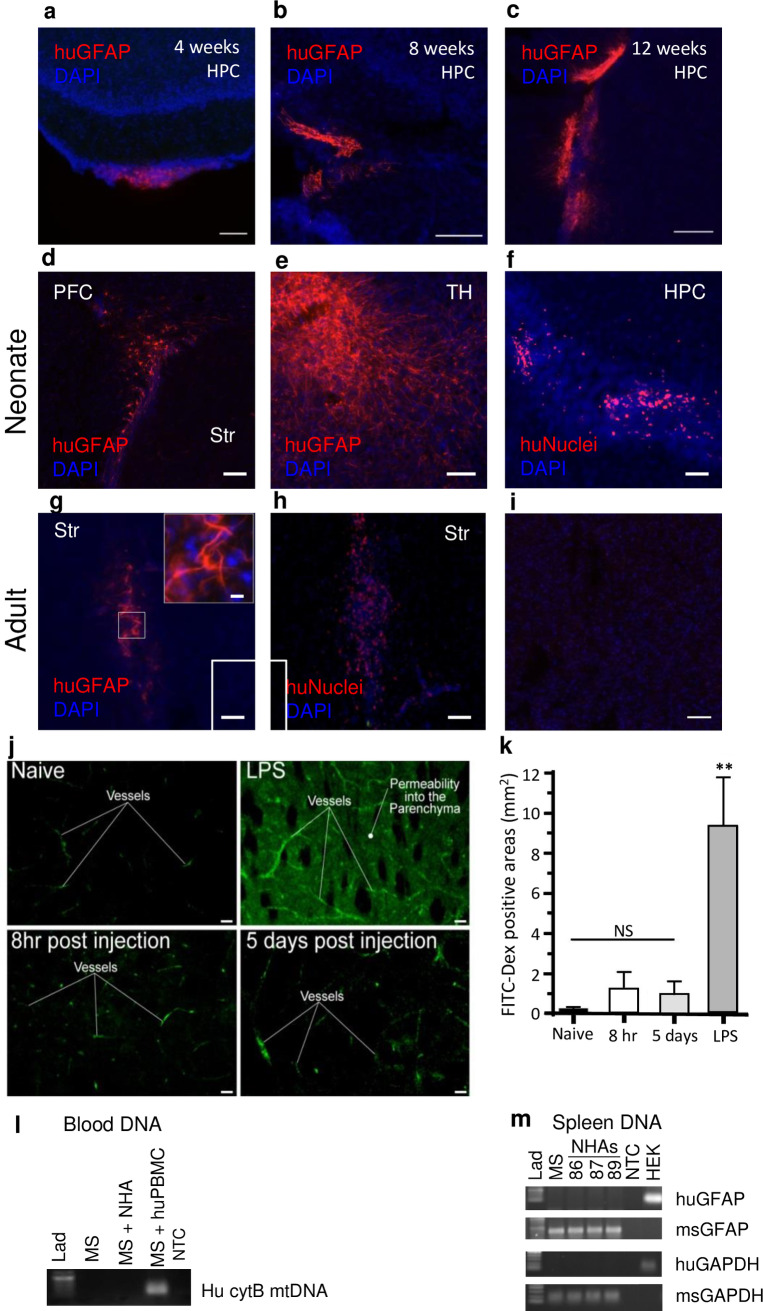
Transplanted astrocytes survive and develop processes in neonate and adult NSG mice but spread out only in neonate NSG mice, without evidence for disruption of blood brain barrier or xenotransplanted astrocyte migration outside of the brain. 200,000 NHAs were injected into neonate brain at postnatal day 1 (PND1) or 50,000 NHAs into each striatum of adult (5–6 weeks) NSG mice. At 4 weeks (**A**), 8 weeks (**B**) and 12 weeks (**C**) post xenotransplantation in neonates, NHA expression is shown by human GFAP (huGFAP, red) with DAPI (blue) staining. Data is representative of 2 mice per time point. Representative images of neonatal prefrontal cortex (PFC, **D**), thalamus (TH, **E**), and hippocampus (HPC, **F**) immuno-stained for human GFAP (huGFAP, red) or human Nuclei (huNuclei, red) and DAPI (blue) at 12 weeks. Representative images of adult striatum immune-stained for human GFAP (**G**, red) or huNuclei (**H**, red) and DAPI (blue) at 4 weeks 5 days post xenotransplantation. Inset: higher magnification of square. (**I**) Representative image of negative staining control for rabbit or mouse IgG primary and corresponding secondary of adult non HIV-infected mouse striatum with DAPI (blue). Data is representative of 2 mice per time point. (**J-K**) Evaluation of cerebrovascular permeability in the striatum following intracranial implantation of astrocytes by microinjection in adult animals. Animals were transcardially perfused with a fluorescence tracer (FITC) conjugated to 10kDa dextrans in the following groups of animals: naïve, 4hrs after exposure to bacterial LPS (*i*.*p* 6mg/kg), 8hrs or 5 days following microinjections of transplanted exogenous astrocytes. **(J)** Representative confocal images of striatal areas at the injection coordinates (relative to Bregma +0.5 A/P, +1.8 M/L, −3.0 D/V). **(K)** Image analysis measurements of vascular permeability using pixel intensity (as a function of area) in the parenchymal areas of the injection site. Results are shown as the average ±SEM; multiple group comparisons were determined by one-way ANOVA with post-hoc Dunnett's test * = p<0.001, *n* = 3–4 per condition, NS, denotes no significance. (**L**) Adult mice were injected with NHAs without huPBMC reconstitution and real-time PCR hu cytoB mtDNA products extracted from the blood within 5 hours of injection were analyzed by electrophoresis. MS: blood from non-NHA injected animal, MS + NHA: blood from animal microinjected with NHAs, MS + huPBMCs: animals reconstituted with huPBMCs, NTC: no template control. (**M**) Adult mice were injected with NHAs without huPBMC reconstitution and real-time PCR DNA products (human GFAP, human GAPDH, mouse GFAP and mouse GAPDH) were analyzed by electrophoresis at day 5 post xenotransplantation. *n* = 3. MS: spleen from 1 non-NHA injected animal, HEK: human HEK293T cells. Scale bars: **A-J**, 50μm; **G** insert, 10μm.

Given the inability for invasive studies to examine the role of the CNS in general and astrocytes in particular as a reservoir for HIV in humans, and to track HIV egress from brain to peripheral organs, we developed a chimeric human astrocyte/human PBMC mouse model to address the role of astrocytes in harboring HIV and in disseminating HIV from the brain to the periphery. We transplanted primary normal human astrocytes (NHAs) or an astrocytoma cell line (U138MG) into the brain of neonate or adult NSG mice. Specifically, 200,000 NHAs were injected into four regions of the brain at post-natal day 1 (PND1) or 50,000 NHAs were microinjected into each striatum of adult (5–6 weeks) mice. Transplanted astrocytes survived and developed processes in neonates and adults, but spread from the injection site only in neonates. At four weeks post transplantation in neonates, NHAs are found in the hippocampus (**Fig 1A**) and striatum as indicated by human glial fibrillary acid protein (huGFAP) and DAPI staining. At this time, they are clustered together and did not have extensive processes. By eight weeks post transplantation, NHAs are more migratory, extended longer processes as shown by huGFAP, and were found in the hippocampus (**Fig 1B**), striatum and around lateral ventricles. By twelve weeks of age, NHAs have an even greater degree of migration (**Fig 1C**). NHAs also demonstrated extensive networks of processes and engraftment into many areas of the brain including but not limited to the prefrontal cortex (**Fig 1D**), thalamus (**Fig 1E**), hippocampus (**Fig 1F**), corpus callosum, and striatum as shown by huGFAP and human nuclei (huNuclei) staining. In adult mice, by five weeks post transplantation, NHAs survived and formed processes although they remained more localized to the site of injection (**Fig 1G and 1H**). Spread of astrocytes in the brain of neonates and not adults was expected given the extensive development that the neonate brain undergoes during this timeframe.

To examine blood brain barrier (BBB) integrity after astrocyte injection, we perfused fluorescent tracer (FITC) conjugated to 10kDa dextran into animals with 1) no injection as a negative control 2) eight hours after adult animals received astrocyte injections into each striatum 3) five days after astrocyte injection, the time point the adult animals were reconstituted with huPBMCs or 4) four hours following LPS (IP 6mg/kg) treatment as a positive control for BBB permeability. As shown in **Fig 1J** and quantified in **Fig 1K**, control animals showed excellent retention of the FITC-dextran tracer whereas the positive LPS control showed significant BBB permeability. Animals that were implanted with astrocytes showed minimal vascular permeability at 8hrs, and no detectable breach to the barrier was observed at 5 days post microinjection of astrocytes. We also could not detect human DNA outside of the CNS in animals not reconstituted with huPBMCs after astrocyte engraftment. Human cytochrome B mitochondrial DNA (hu cytB mtDNA), which is specific to humans and has thousands of copies per cell [41,42], was not present in the blood of animals with exposure to no human DNA (MS) or, importantly, animals within hours of NHA injection (MS + NHA). As expected, hu cytB mtDNA was present in reconstituted animals (MS + huPBMC) (see **Fig 1L**). Further, engrafted astrocytes were not detected outside of the CNS four weeks after injection as evaluated by lack of detection of human GAPDH or human GFAP DNA in the spleen of animals transplanted with NHAs without huPBMCs reconstitution (**Fig 1M**). Subsequent studies were performed in parallel in both neonate and the adult model.

### HIV infected astrocytes support HIV replication in vivo and egress of HIV from the brain to peripheral tissue

**Fig 2:**
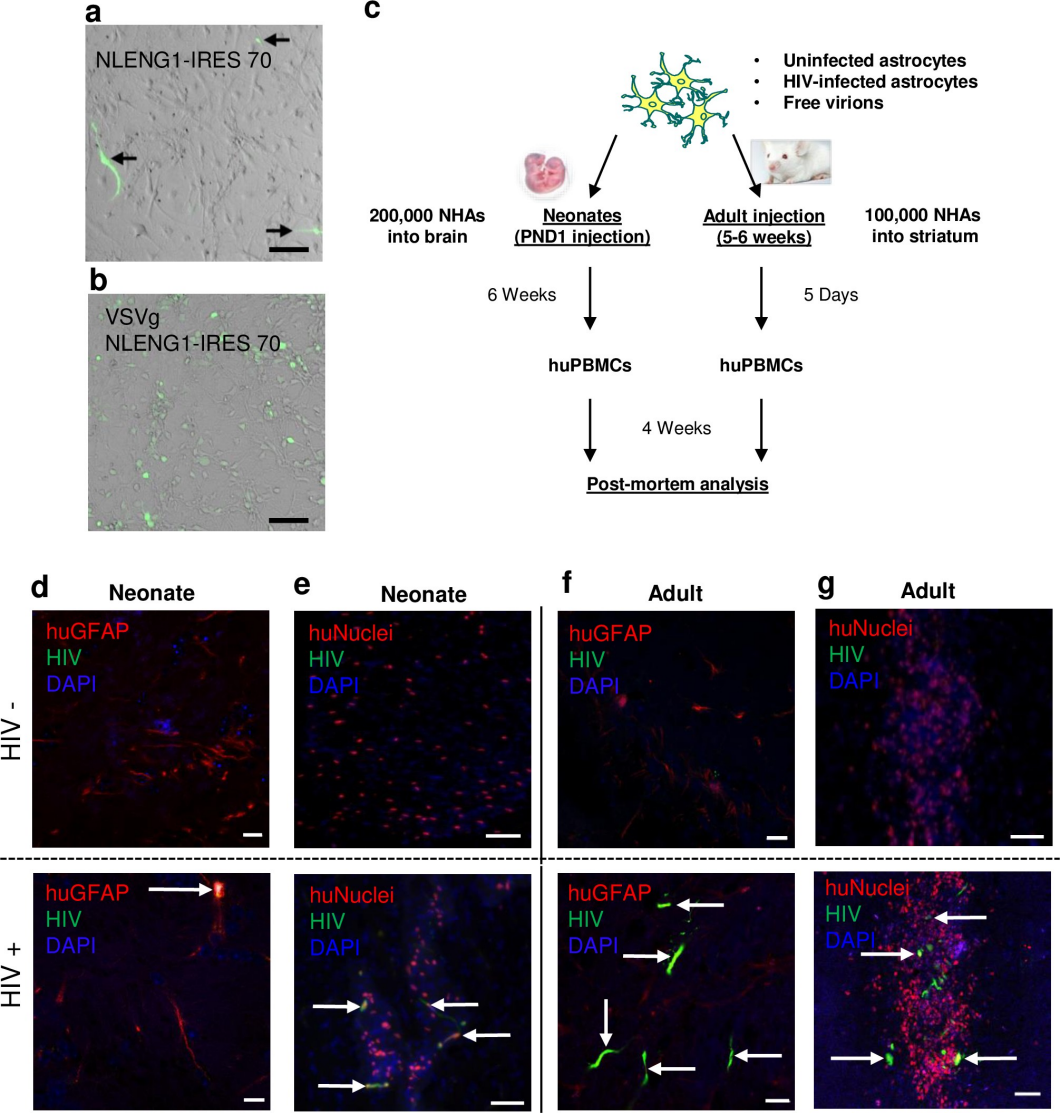
HIV in the brain of neonate and adult NSG mice xenotransplanted with astrocytes. NHAs were infected with GFP-expressing full length HIV virus (**A**, NLENG1-IRES 70, 1–3% GFP+ cells), or with HIV_VSVg_ pseudotyped (**B**), VSVg-NLENG1-IRES 70, 20–30% GFP+ cells), both viruses express GFP. (**C**) Schematic of experimental design. HIV- or HIV+ NHAs were injected into mouse brain on PND1 (200,000 total NHAs) or adult striatum (5–6 weeks, 100,000 total astrocytes). At six weeks post PND1 xenotransplantation or 5 days post adult xenotransplantation, mice were reconstituted with huPBMCs. At four weeks post reconstitution animals were sacrificed for post-mortem analyses. Representative images of neonates (**D-E**) or adult (**F-G**) mice injected with NHAs or NHAs infected with HIV. (**D**) Representative images of neonate mice xenotransplanted with HIV- (top) or HIV+ (bottom) NHAs. Arrow indicates co-localization of huGFAP (red) and HIV (green) along with DAPI (blue) in mouse striatum. (**E**) Representative image of neonates xenotransplanted with NHAs (top) and NHA-HIV (bottom) for huNuclei (red), HIV (green) and DAPI (blue). Arrows indicate co-localization of huNuclei and HIV. (**F**) & (**G**) Representative images of adults injected with HIV- (top) or HIV+ (bottom) NHAs for either (**F**) huGFAP (red), HIV (green) and DAPI (blue) or (**G**) huNuclei (red), HIV (green) and DAPI (blue). Arrows indicate co-localization of human markers and HIV. Scale bars: **B-C, E, G**, 50μm; **D**, **F**, 10μm.

We next determined whether HIV infected astrocytes harbor replication-competent HIV in vivo to allow for HIV egress from the brain to peripheral tissue. NHAs were infected in vitro with GFP-expressing full length HIV virus (NLENG1-IRES 70), with or without vesicular stomatitis virus G (VSVg) pseudotyping. Rate of HIV infection in astrocytes was 1–5% or 20–30% based on GFP expression from wild-type (**Fig 2A**) or VSVg pseudotyped (**Fig 2B**) virions respectively. Post-mortem analysis of rate of HIV infection in astrocytes is consistent with NHA infection rate based on previous studies [32,34]. HIV-infected or uninfected NHAs were then injected into the neonatal or adult mouse brains, the animals were reconstituted with huPBMCs except where stated and sacrificed four weeks later as depicted in **Fig 2C**. By 10 weeks (neonatal model; **Fig 2D and 2E**) or 5 weeks (adult model, **Fig 2F and 2G**) post injection, HIV-uninfected and infected NHAs survived as shown by GFP-huGFAP or GFP-huNuclei co-expression in neonate and adult mice. Evidence of HIV infected astrocytes was visualized by co-localization of huGFAP and HIV-GFP (**Fig 2D and 2F**) and huNuclei and HIV-GFP (**Fig 2E and 2G**). Further, adult mice injected with HIV-infected NHAs at one, five and 33 days post transplantation (without huPBMC reconstitution) demonstrated presence of HIV DNA in the brain with no change in levels over time. HIV RNA trended towards a decrease over time, although not significantly.

### Transplantation of VSVg-pseudotyped HIV-infected astrocytes into neonate or adult NSG mice supported HIV egress from the brain to peripheral tissue

**Fig 3:**
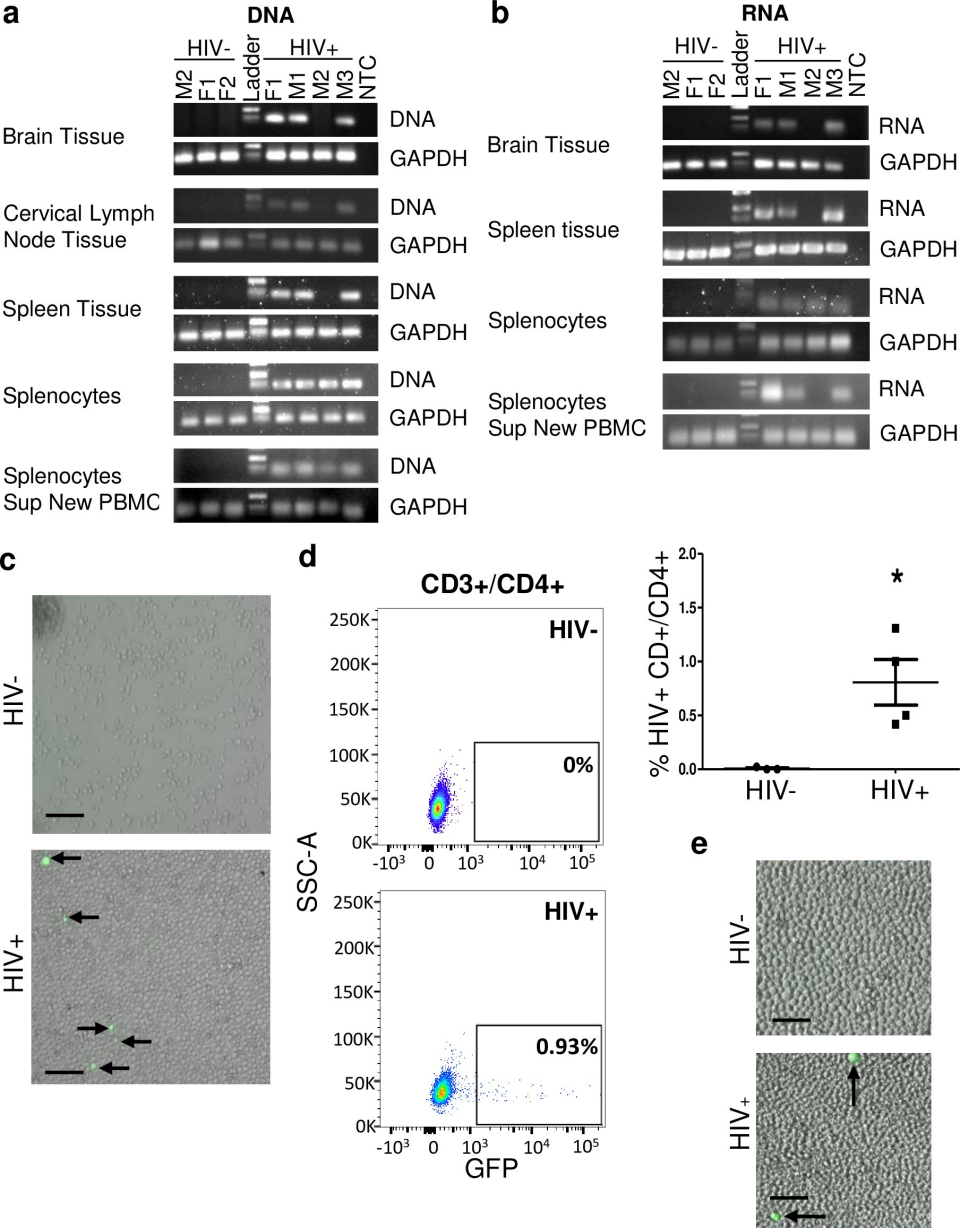
HIV egress from the brain into the periphery in the neonate astrocyte xenotransplantation model. Neonate NSG mice were xenotransplanted with HIV- or HIV+ NHAs as described in Fig 2C and HIV detection outside of the brain was evaluated by Real-time PCR products (HIV DNA, **A**, or HIV RNA, **B**) from brain, cervical lymph node, spleen, splenocytes outgrowth assay and splenocytes outgrowth assay supernatant added to fresh PBMCs analyzed by electrophoresis and human GAPDH. Each column indicates individual animal. HIV- animals are shown to left of ladder, HIV+ animals shown to right for all gels shown. No template control (NTC) is the negative control. M/F indicates sex and number is animal number from that group. (**C**) GFP expression in splenocytes at day 14 day of culturing the cells and (**D**) flow cytometry of cultured splenocytes stained for CD3+/CD4+/GFP; dot blots (left) and cumulative data on right (*p* = 0.05, Mann-Whitney U-test). (**E**) Representative image of supernatant from splenocytes cultured for 14 days from neonates from HIV- animal (top) or HIV+ animal (bottom) on fresh PBMCs stimulated with soluble α-CD3 and α-CD28 IL-2. Egress for neonates was analyzed in *n* = 7 (HIV-) and 12 (HIV+) mice; *n* = 3 (HIV-) and 4 (HIV+) representative mice are shown here; *n* = 3 (HIV-) and 5 (HIV+) representative mice are shown here.

**Fig 4:**
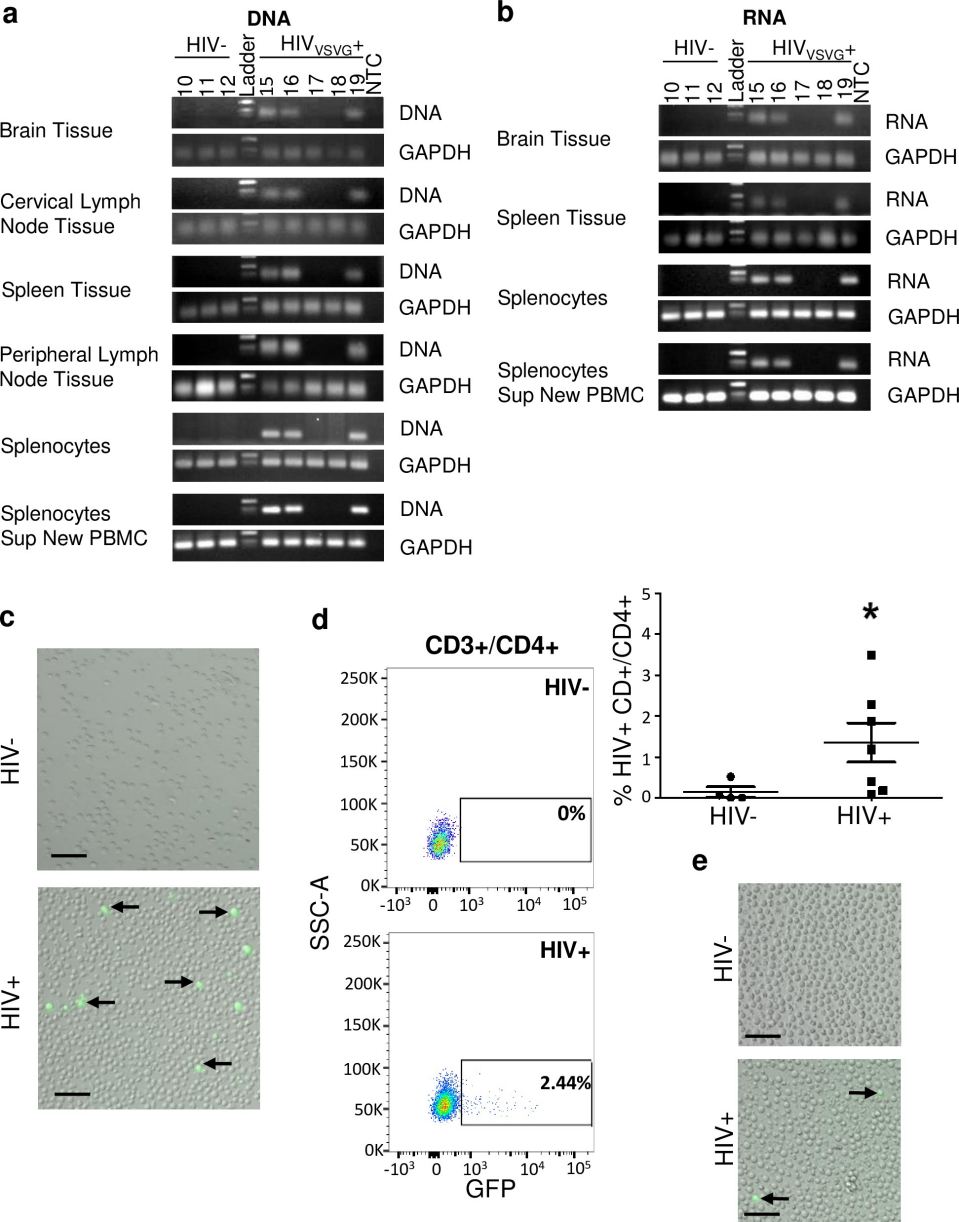
HIV egress from the brain into the periphery in the adult astrocyte xenotransplantation model. Adult NSG mice were xenotransplanted with HIV- or HIV+ NHAs as described in Fig 2C and HIV detection outside of the brain was evaluated by Real-time PCR products (HIV DNA, **A**, or HIV RNA, **B**) from brain, cervical lymph node, spleen, peripheral lymph node, splenocytes outgrowth assay and splenocytes outgrowth assay supernatant added to fresh PBMCs analyzed by electrophoresis and human GAPDH. Each column indicates individual animal. HIV- animals are shown to left of ladder, HIV+ animals shown to right for all gels shown. No template control (NTC) is the negative control. (**C**) GFP expression in splenocytes at day 14 day of culturing the cells and (**D**) flow cytometry of cultured splenocytes stained for CD3+/CD4+/GFP; dot blots (left) and cumulative data on right (*p* = 0.04, Mann-Whitney U-test). (**E**) Representative image of supernatant from splenocytes cultured for 14 days from neonates from HIV- animal (top) or HIV+ animal (bottom) on fresh PBMCs stimulated with soluble α-CD3 and α-CD28 IL-2. Egress was analyzed in *n* = 7 (HIV-) and 9 (HIV+) mice; *n* = 3 (HIV-) and 5 (HIV+) representative mice are shown here.

In neonates (**Fig 3**), within four weeks post reconstitution, HIV DNA and RNA were detected in the brain and peripheral organs including spleen and lymph node as measured by quantitative TaqMan real-time PCR (**Fig 3A and 3B**). Splenocyte outgrowth assay demonstrated the presence of GFP expressing (HIV infected) cells in culture (**Fig 3C**). Flow cytometry analysis of cultured splenocytes identified the GFP+ cells as predominately CD4+ T lymphocytes (**Fig 3D**). Supernatant from the splenocyte outgrowth assay added to freshly isolated PBMCs demonstrated presence of GFP+ cells indicating HIV infection (**Fig 3E**). Similar results were observed in the adult model (detection of HIV DNA/RNA in brain, spleen, and cervical lymph nodes, detection of HIV via splenocyte outgrowth assay, infection of freshly isolated PBMCs by supernatant of splenocytes (**Fig 4A–4E**). Plasma viral load measured in adult xenotransplanted astrocyte animals had a broad range though averaged over one million copies/ml and is likely a reflection of on-going HIV replication in peripheral organs post initial egress from the CNS. Real-time PCR graphs of HIV DNA and RNA from peripheral organs are found in **S1 and S2 Figs.** Together, these studies demonstrate that HIV-infected astrocytes release productive HIV from astrocytes which can egress from the CNS and into peripheral organs.

### Xenotransplanted human astrocytes infected with non-pseudotyped HIV also demonstrate HIV egress into peripheral organs

**Fig 5:**
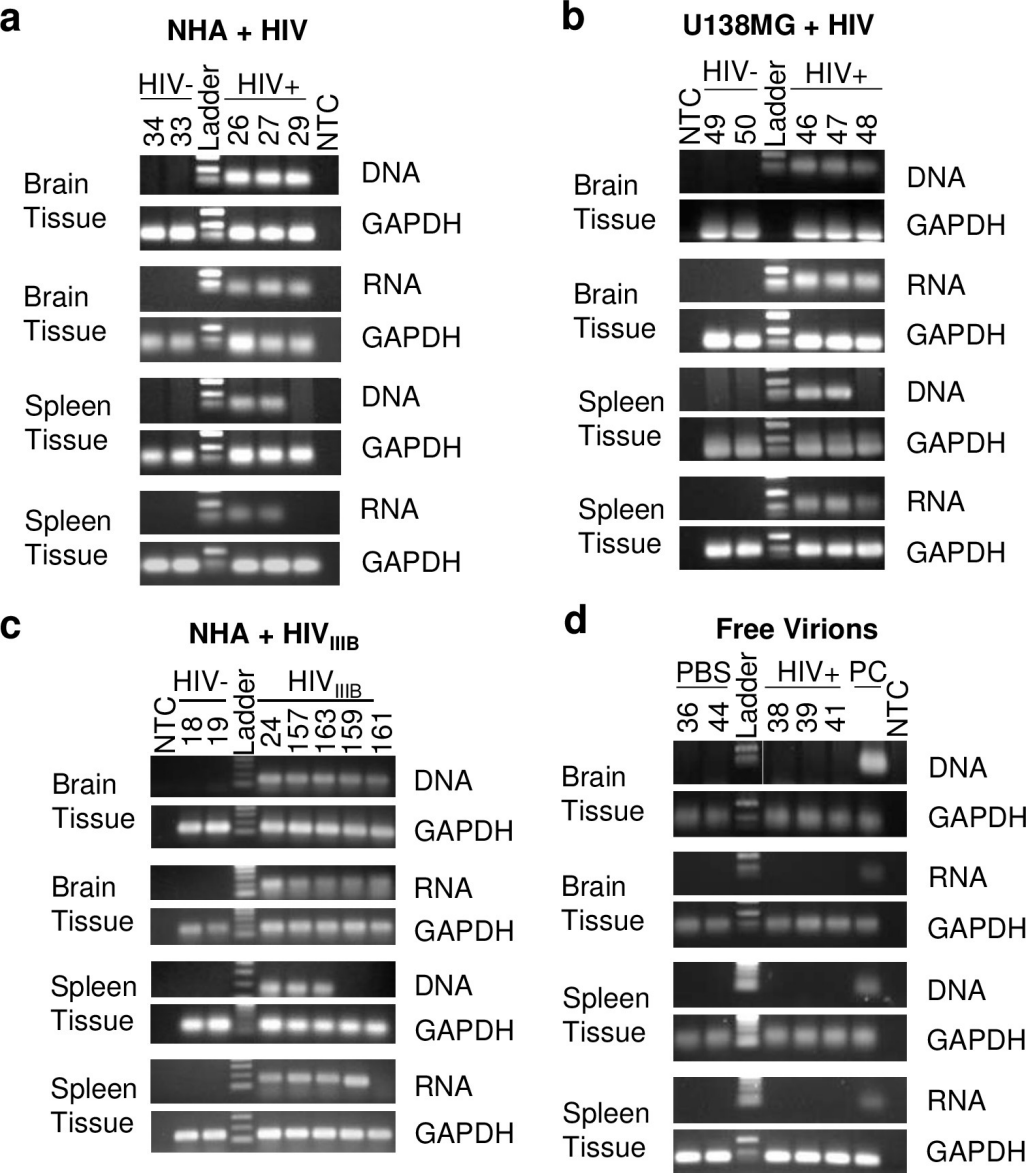
HIV egress from the brain into the periphery in the adult astrocyte xenotransplantation model. Adult NSG mice were xenotransplanted with HIV- or HIV+ astrocytes as described in Fig 2C and HIV detection outside of the brain was evaluated by electrophoresis. (**A**) HIV DNA and RNA from the brain and the spleen of *n* = 2 (HIV-) and 3 (HIV+) adult animals xenotransplanted with HIV- or HIV+ (non-pseudotyped) NHAs. (**B**) HIV DNA and RNA from brain and spleen from *n* = 2 (HIV-) and 3 (HIV+) adult animals xenotransplanted with HIV- or HIV_VSVg_+ U138MG astrocytoma cell line. (**C**) HIV DNA and RNA from brain and spleen from *n* = 2 (HIV-) and 5 (HIV+) adult animals xenotransplanted with HIV- or HIV_IIIB_+ NHAs. (**D**) HIV DNA and RNA from brain and spleen from *n* = 2 (HIV-) and 7 (HIV+) adult animals injected with HIV- or HIV_VSVg_+ free virus into the striatum in the absence of astrocytes. PC indicates a Positive Control for HIV DNA and HIV RNA.

Given that VSVg pseudotyping is not endogenous to HIV virus but used to increase viral infection of NHAs, we repeated the experiment by infecting astrocytes with non-pseudotyped infectious HIV which were then xenotransplanted into NSG mice and HIV in peripheral organs was evaluated post immune reconstitution. As shown in **Fig 5A**, mice positive for HIV in the brain were also positive for HIV DNA and RNA in the spleen. To exclude that this phenomenon is not specific to neonatal astrocytes, we repeated the experiment using an adult human astrocytoma cell line U138MG, which also led to detection of HIV DNA and RNA in brain and spleen (**Fig 5B**). To exclude that this phenomenon is not specific to the genetically-engineered HIV-1 NLENG1-IRES 70 strain, NHAs were infected with patient-derived HIV-1 IIIB strain and injected into adult animals. These animals also demonstrated HIV egress from the CNS to periphery as shown by the presence of HIV DNA and RNA in the brain and spleen (**Fig 5C**). Another possibility for HIV detection in the periphery is the escape of virus (free or loosely bound to astrocyte cell membrane) at the time of astrocyte transplantation into circulation and subsequent infection of CD4+ T cells in spleen and lymph nodes post huPBMC reconstitution. Although this possibility is remote especially in the case of the neonate model where there is a six weeks lag between injection of HIV-infected astrocytes in the brain and huPBMC reconstitution, we nonetheless addressed this possibility by injecting free virus (1-2ng of p24/hemisphere) into the striatum of adult animals in the absence of astrocytes. Reconstitution took place five days later as with other adult injections. No animals injected with free virus had detectable HIV DNA or RNA in any organ tested (**Fig 5D**). Real-time PCR graphs are found in **S3 Fig**. Collectively, these studies demonstrate that HIV infection initiated in astrocytes can support in vivo productive HIV replication/release to allow for HIV egress from the brain to peripheral organs.

### Evidence of HIV evolution in peripheral organs initiated from HIV infected astrocytes in vivo

**Fig 6:**
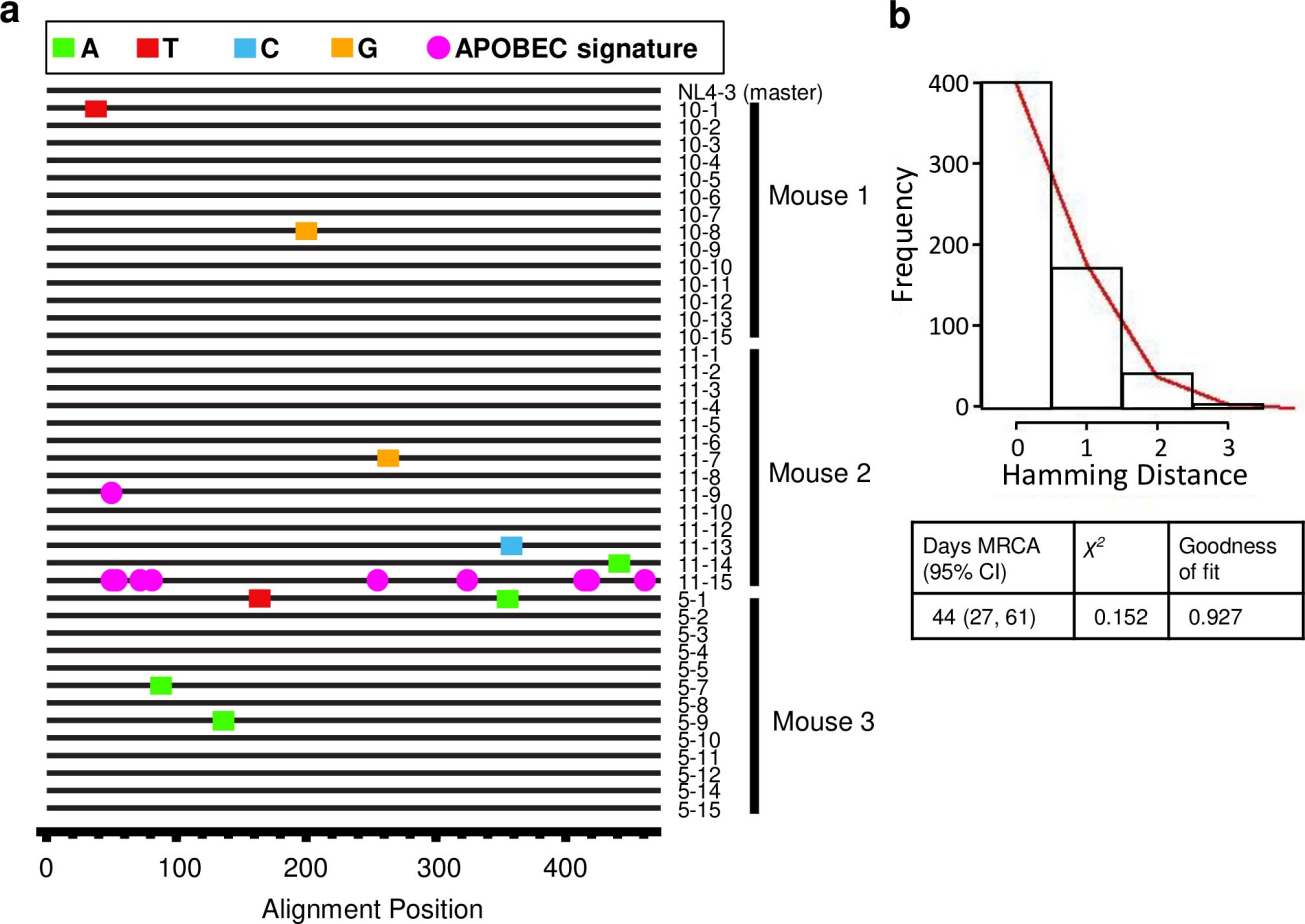
HIV evolution in NSG mice. (**A**) Highlighter plot denoting the locations of nucleotide substitutions in each *gag* sequence from 3 mice xenotransplanted with HIV-infected astrocytes (horizontal lines) in comparison to the virus inoculum (NL4-3, top sequence). Nucleotide substitutions are shown as color-coded tick-marks, and APOBEC-signature mutations are shown as pink circles. (**B**) Hamming distance plot demonstrating Poisson distribution and estimated days to most recent common ancestor (MRCA) with supporting statistics are shown. Actual days to MRCA (days post infection) were 33. Scale bars: 50μm.

To assess viral evolution within this model, we performed single genome amplification (SGA) of HIV *gag* from three adult animals xenotransplanted with astrocytes infected with HIV (NLENG1-IRES /non-pseudotyped). Splenic DNA was collected 33 days post-xenotransplantation and subjected to serial dilution until single viral genomes were amplified and directly sequenced, as described [43]. Analyzing a 473 bp region of *gag*, we found that in a total of 41 single genome sequences (13–14 from each animal), 9 (22%) had point mutations from the original viral inoculum (NL4-3). The remaining 32 were identical to original (parent) HIV, as demonstrated by highlighter plot (**Fig 6A**). The mutated genomes show random diversification, as observed in HIV-1 infected patients with the same duration of infection [44]. Statistical analysis showed that the virus population exhibited Poisson distribution and star-like phylogeny using a goodness of fit test [45], and time to most recent common ancestor (MRCA) analysis correctly identified the duration of infection (95% confidence intervals = 27–67 days, actual = 33 days) (**Fig 6B**), suggesting that evolution observed in this mouse model fits the mathematical model of continual HIV-1 replication and diversification determined in humans. Of note, this data only reflects the rate of HIV evolution in the mice, and not the cellular source of the evolved virus. One sequence shows evidence of extensive APOBEC hypermutation, with 9 APOBEC-signature mutations in this 473 nt range, which is also commonly observed in acute human HIV-1 infection. Thus, this model mimics early HIV-1 virus evolution observed in humans, even in the absence of immune selection pressure (expected given RT infidelity) and most importantly, detection of these mutations indicates that virus initiated from astrocytes can continue to replicate and evolve/mutate to mimic the viral evolution observed in early peripheral infection and potentially can contribute to peripheral genetic diversity.

### Depleting PBMCs of both CD4+ T cells and monocytes prior to reconstitution of HIV infected xenotransplanted astrocytes in NSG mice significantly reduces HIV egress into peripheral organs

**Fig 7:**
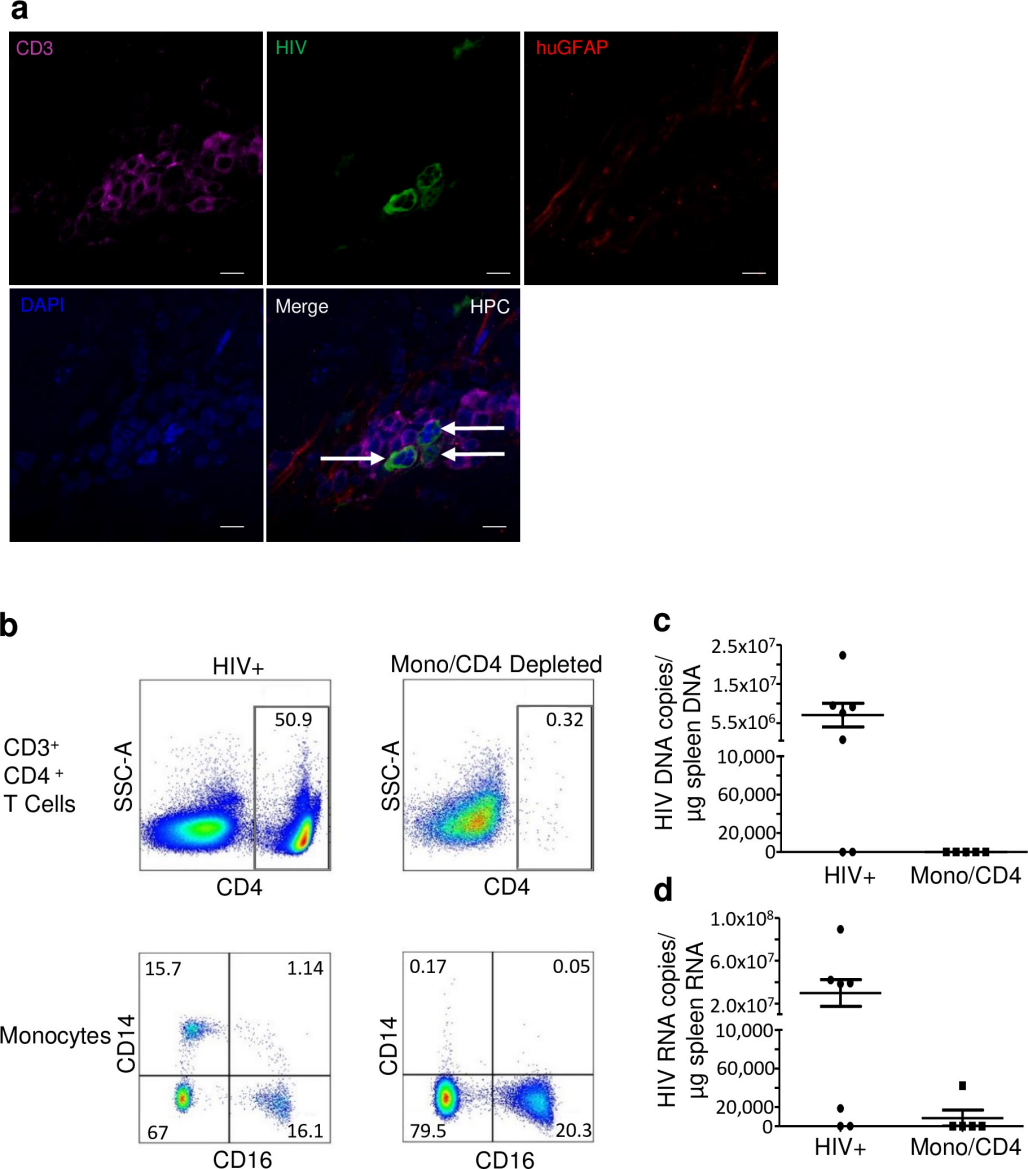
HIV egresses from the brain into the periphery likely through CD4+ T cells. (**A**) Representative image of neonate hippocampus (HPC) immunostained for human T cells (CD3+, magenta), human astrocytes (huGFAP; red), HIV (green) and Nuclei (DAPI, blue) at week 10 post-xenotransplantation and week 4 reconstitution with huPBMCs. Arrows indicates detection of CD3+/HIV+ cell. Scale bar, 10μm. (**B**) Total PBMCs (HIV+) or monocyte/CD4+ T cell depleted PBMCs analyzed by flow cytometry for CD3+/CD4+ positive T cells (top) or CD14 positive or CD14/CD16 positive monocytes before injection into the adult mouse xenotransplanted with HIV_VSVg_ + NHAs. HIV DNA (**C**) and RNA (**D**) from spleen from animals xenotransplanted with HIV_VSVg_+ NHAs and reconstituted with total PBMCs (HIV+) or PBMCs depleted of monocytes and CD4+ T cells (Mono/CD4). No analysis was performed on DNA as Mono/CD4 group had undetectable HIV DNA. RNA is depicted on the right (*p = 0*.*08*, Mann-Whitney U-test). *n* = 5–7 per group.

To address how HIV from infected astrocytes can egress to peripheral organs, we evaluated the brain of NSG-Astro-HuPBMCs mice for evidence of infected lymphocytes in the brain post immune reconstitution of the animals. We demonstrate the presence of CD3+/HIV+ T cells in the hippocampus of xenotransplanted HIV infected astrocytes post reconstitution with PBMCs (**Fig 7A**). Next, we depleted PBMCs of monocytes and CD4+ T cells using RosetteSep to achieve greater than 99% monocyte/CD4+ T cell depleted PBMCs (**Fig 7B**) then reconstituted astrocyte-transplanted adult mice. We found that monocyte/CD4+ T cell depletion led to undetectable HIV DNA in all animals (**Fig 7C**) and undetectable RNA in 4 out of 5 animals in the spleen (**Fig 7D**). These findings suggest that HIV egress from the brain to peripheral organs occurs through trafficking of immune cells, most probably T cells and not monocytes because although monocytes become macrophages as they enter tissues, including the brain, once they are tissue macrophages they may migrate within a tissue but there is no evidence (outside of metastasis) that macrophages can migrate out of tissue and back into circulation. As such, it is more likely that trafficking of infected T cells is responsible for HIV egress out of brain in our model.

### HIV infected astrocytes support low levels of HIV egress under cART

**Fig 8:**
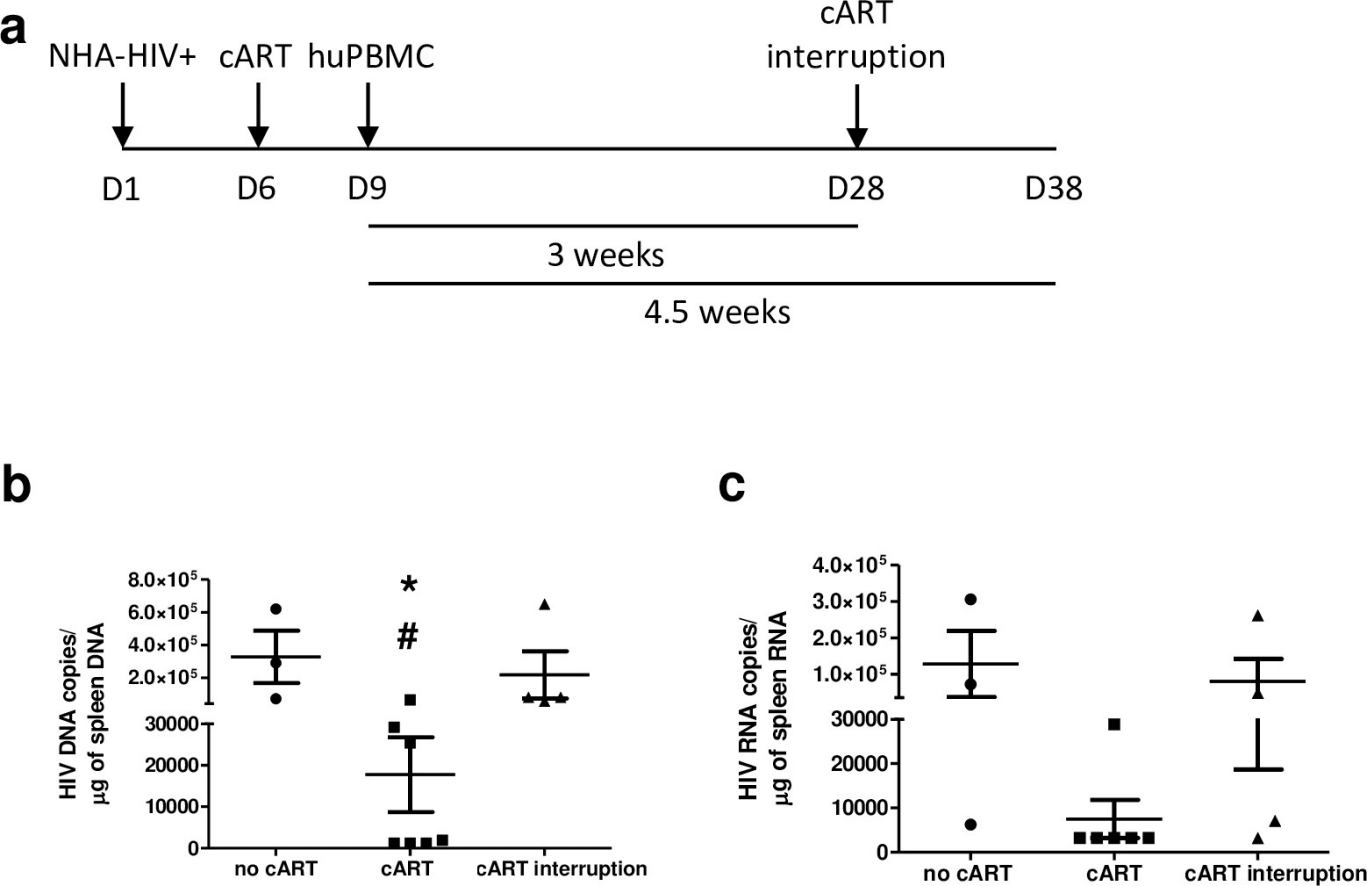
Astrocyte derived HIV egress under cART. (**A**) Adult NSG mice were xenotransplanted with HIV+ astrocytes and treated with cART as depicted and cART treatment continued every other day until sacrifice at 3 or 4.5 weeks post reconstitution. cART interrupted animals were given 3 weeks of cART followed by 1.5 weeks of interruption before sacrifice. (**B**) HIV DNA was measured in the spleen by electrophoresis at time of sacrifice; with Kruskal-Wallis comparison (H = 9.185, *p* = 0.01) with Dunn post hoc test. * *p < 0*.*05* from no cART, # *p < 0*.*05* from cART interruption. HIV RNA was measured from spleen and shown in **C;** with Kruskal-Wallis test comparison (H = 5.904, *p* = 0.052). Data combined for three and four-and-a-half-week cART treatments as there were no statistical differences. *n* = 3–4 per group.

To assess the contribution of astrocytes to HIV egress under cART, HIV infected astrocytes were injected into the adult mouse brain. Five days later, cART (tenofovir 211mg/kg; emtricitabine 205mg/kg; raltegravir 56mg/kg) or vehicle treatments were initiated and continued until sacrifice. Three days following cART initiation, animals were reconstituted with huPBMCs (**Fig 8A**). HIV DNA levels in the spleen were significantly reduced (**Fig 8B**). This data demonstrates that astrocyte supports ongoing HIV egress, albeit to a lower extent, under cART. This may be driven by lower cART penetration into the CNS.

To model HIV rebound after cART interruption, animals were injected with HIV+ astrocytes, began cART treatment five days later and three days after that were reconstituted with huPBMCs. Animals were treated with cART for three weeks after reconstitution then cART was interrupted for ten days till sacrifice. Rebound viral DNA and RNA were detected in the spleen at time of sacrifice (**Fig 8B and 8C**). These results indicate that astrocytes release low levels of HIV even under cART administration, albeit cART penetration into CNS and other tissue is less than in periphery. Following cART interruption virus rebound is likely due to new rounds of peripheral infection.

### Astrocytes are infected through systemic HIV infection

**Fig 9:**
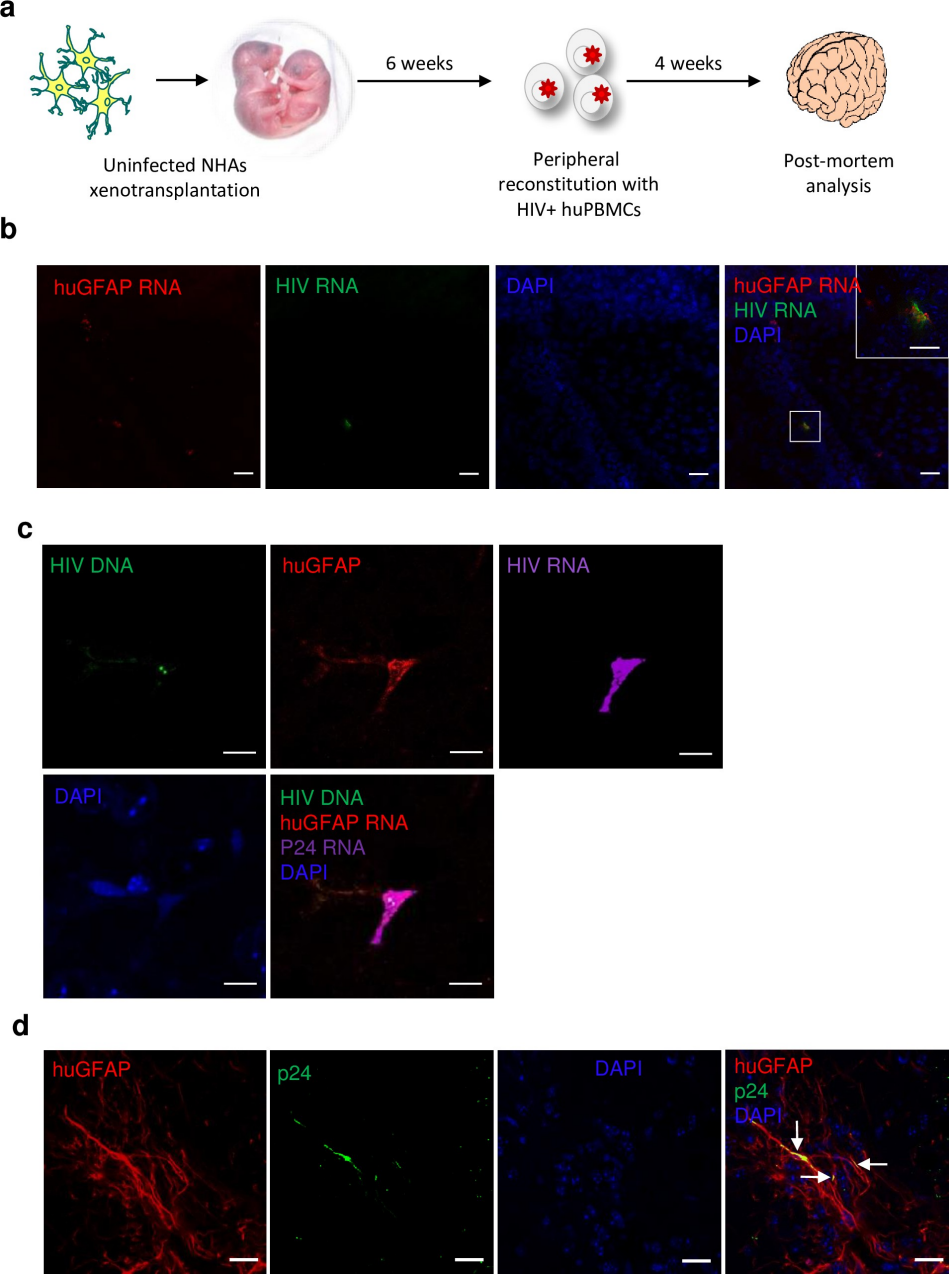
Evidence of HIV infection of astrocytes through systemic route of infection. (**A**) Neonatal mice were xenotransplanted with uninfected NHAs and reconstituted with HIV+ huPBMCs and sacrificed 4 weeks later. (**B**) Representative image of neonate striatum of co-localization of human GFAP RNA (red) HIV RNA (green) and DAPI (blue) by RNAscope. (**C**) Representative image of neonate hippocampus with HIV DNA (green), huGFAP (red), HIV RNA (purple) and DAPI (blue). (**D**) Representative image from neonate hippocampus immunostained for human astrocytes (huGFAP; red), HIV p24 (green) and Nuclei (DAPI, blue). Arrows indicate co-localization of huGFAP and p24. *n* = 6 mice. 4 or 6 coronal sections were analyzed per mouse for RNAscope and immunofluorescence, respectively. Representative images chosen from 4 of 6 mice with observed HIV infection, with 1–4 infection events observed per animal. B, D scale bar, 20μm. **B** insert, 10μm. **C** scale bar, 5 μm.

Our model relied on transplanting the mice with ex vivo HIV infected astrocytes. To assess whether HIV in the periphery can infect astrocytes in vivo, we injected neonate mice with uninfected astrocytes then reconstituted the animals with HIV-infected human PBMCs at 6 weeks (**Fig 9A**); the majority of HIV+ cells are CD4+ T cells (94%) with a smaller percentage of CD14+ monocytes. Of note, in our ex vivo HIV-infected human PBMC culture system monocytes quickly adhere to the plate and we use the non-adherent cells to infect which are predominately T cells. Four weeks post reconstitution; the animals were sacrificed. Through this route of systemic infection, we show presence of HIV+ astrocytes, as determined by HIV and huGFAP co-localization using both RNAscope and immunofluorescence. Specifically, human GFAP RNA and HIV RNA are co-localizing in the striatum as shown in **Fig 9B** and in **Fig 9C** we show HIV DNA, RNA and huGFAP immunofluorescence co-localization in the hippocampus of a different neonate than that from **Fig 9B**. **Fig 9D** (additional images are shown in **S4 Fig**) shows co-localization of huGFAP and p24 immunofluorescence in yet another neonate in the hippocampus. These findings indicate that astrocytes are infected in vivo under a natural route of HIV neuroinvasion (e.g. from periphery to CNS). To assess the ability of cART to block astrocytic HIV infection we xenotransplanted HIV- human astrocytes into neonates and began cART treatment one day prior to and every other day post HIV+ human PBMC reconstitution. In animals treated with cART we found no evidence of astrocyte HIV infection (**S5 Fig**) suggesting a normal course of infection of astrocytes in vivo. It is important to note that while this suggests cART prevents new astrocytic infection, it would not block astrocyte infection due to the initial invasion of HIV into the CNS up until cART initiation. There was no evidence of human astrocyte and CD3 co-expression with or without the presence of HIV suggesting that astrocytes are infected with HIV and is not merely a consequence of astrocyte phagocytosis of T cells.

### HIV detection in astrocytes from human brain of donors under cART

**Fig 10:**
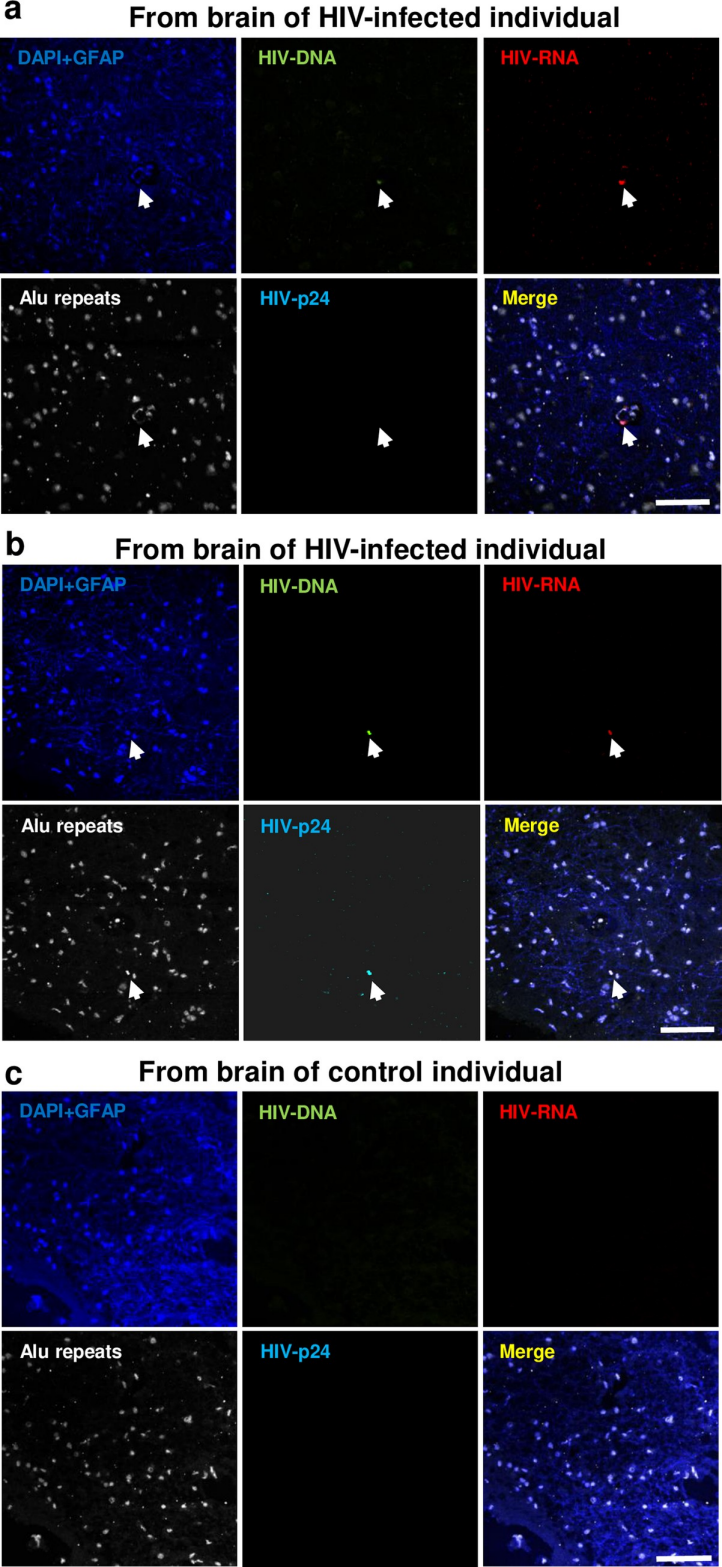
HIV+ astrocytes detected in human brain of peripherally suppressed donors. HIV+ astrocytes are detected in cortical and hippocampal brain tissue sections obtained from HIV infected individuals under effective ART viral suppression. Staining for these human tissue sections was performed for DNA (DAPI, blue), GFAP (Alexa 350, blue), Alu-repeats (probe for Alu, white), HIV-nef DNA (green), HIV-mRNA (red), and HIV-p24 protein (cyan). To separate all these fluorescent sequential scanning and spectral detection was used as well as each respective control. In addition, co-localization of DAPI, Alu-repeats, and HIV-DNA was considered a positive nuclear signal. In contrast, HIV-mRNA-gag, GFAP, and HIV proteins had minimal to no co-localization with nuclear markers. (**A** and **B**) Corresponds to a representative positive for HIV-DNA in GFAP positive astrocytes (see arrow). (**C**) No unspecific staining for HIV-nef DNA, HIV mRNA or viral proteins was detected in uninfected tissues. Data is quantified in Table 1. Scale bars: 80μm.

To determine whether astrocytes are infected in humans under effective cART, brain tissue sections were obtained from four HIV infected individuals with suppressed virus and four uninfected individuals (Table 1) then stained for DNA (DAPI, blue staining), GFAP (Alexa 350, Blue staining), Alu-repeats (probe for Alu, white staining), HIV-nef DNA (green staining), HIV-mRNA (red staining), and HIV-p24 protein (Cyan staining). To separate all these fluorescent sequential scanning and spectral detection was used as well as each respective control. In addition, co-localization of DAPI, Alu-repeats, and HIV-DNA was considered a positive nuclear signal. In contrast, HIV-mRNA-gag, GFAP, and HIV proteins had minimal to no co-localization with nuclear markers. Using the approach described above, we evaluated the rate of HIV integrated DNA, HIV-mRNA and HIV-p24 expression in cortical and hippocampal astrocytes (GFAP+ positive cells). We show that GFAP positive astrocytes from HIV infected individuals have between 1.6x10^4^–2.22x10^4^ integrated HIV gag DNA/10^6^ GFAP positive astrocytes captured by laser microdissection. Further, using in situ RNAscope and staining analysis, we found that approximately 2–7% of GFAP+ cells are HIV gag mRNA positive and 1–5% are HIV-p24 positive (Table 1). Representative images from two HIV+ brains (**Fig 10A and 10B**) and a control brain (**Fig 10C**) are shown. This finding is especially remarkable given that our mouse model with less than 5,000 infected astrocytes, a significantly lower infection rate than that measured in humans, was sufficient to support HIV egress from the brain to the periphery.

**Table 1:** Human post mortem analysis of HIV+ astrocytes. Post mortem analysis from cortical or hippocampal brain regions of HIV- or HIV+ individuals. Laser capture microdissection (LCM) of GFAP+ cells analyzed for Alu-gag HIV by PCR. RNAscope and staining determined GFAP+ co-localization with gag mRNA, nef DNA and p24. Staining for each patient included analysis of 12 different sections with 20–24 fields per section (~240 fields) from an average tissue size of 1.5±0.75x0.91±0.66 cm. M: male; F: Female; N.A.: not applicable; U.D.: undetectable. Anti-retrovirals: ATV, atazanavir (Reyataz); EFV, efavirenz (Sustiva); RTV, ritonavir (Norvir); TFV, PMPA; tenofivir DF (Viread); 3TC, lamivudine (Epivir); D4T, stavudine (Zerit FTV, SQV2 or FTV saquinavir-sgc (Fortovase);SQV, saquinavir (Invirase); DDI, didanosine (Videx); NFV, nelfinavir (Viracept). Representative staining images of data are shown in **Fig** 9.

Sample	HIV status	Age	Sex	ART	CD4 counts (cells/mm)	Plasma Viral Load (copies/ml)	CSF Viral Load (copies/ml)	LCM of GFAP+ cells for Alu-Nef HIV DNA/10^6^	% of GFAP+ cells for HIV gag mRNA	% of GFAP+ cells for HIV p24
1	-	69	M	N.A.	N.A.	N.A.	N.A.	-	U.D.	U.D.
2	-	38	M	N.A.	N.A.	N.A.	N.A.	-	U.D.	U.D.
3	-	51	F	N.A.	N.A.	N.A.	N.A.	-	U.D.	U.D.
4	-	54	M	N.A.	N.A.	N.A.	N.A.	-	U.D.	U.D.
5	+	56	M	ATV, EFV, RTV, TFV	421	U.D.	U.D.	1.6x10^4^	2.36±1.89	2.99±1.54
6	+	52	M	N.R.	630	40	<20	3.75x10^3^	3.69±3.05	4.56±2.68
7	+	42	M	3TC, D4T, FTV, SQV	389	U.D.	<20	5.25x10^4^	6.91±2.68	4.25±1.25
8	+	44	M	D4T, DDI, NFV	436	U.D.	U.D.	2.22x10^4^	2.89±1.66	1.22±0.99

## Discussion

HIV latency and/or residual low-level HIV replication is a major obstacle towards an HIV cure. cART intensification alone has not been able to eliminate the latent reservoir pool [1,46]. Much attention is focused on the role of CD4+ resting T cells in HIV latency [4]. However, other cellular reservoirs and sanctuary sites for HIV remain [8]. Considerable debate regarding the role of the brain in general and astrocytes in particular as a reservoir for HIV exists [47]. Our data indicates that astrocytes, the most abundant cell type in the brain and the only source of HIV in our model, harbor HIV DNA/RNA in vivo and that HIV from infected astrocytes egresses, likely through trafficking of CD4+ T cells, into peripheral organs, as indicated by detection of HIV DNA/RNA in spleen and lymph nodes and viral outgrowth assays. This phenomenon occurred with a relatively small number of infected astrocytes (<5000), mimicking the reported low-frequency of HIV infection of astrocytes in post-mortem tissue [31,32,34]. We further demonstrate that HIV+ human brains show evidence for integrated HIV gag DNA and mRNA in 0.4–5.2% and 2–7% of astrocytes, respectively, in patients under cART. Most significantly, the ability of HIV to egress from the brain to the periphery demonstrates that this sanctuary site is not an isolated viral reservoir but rather one that can contribute to ongoing HIV evolution in peripheral organs. Indeed, we demonstrated that HIV originating from astrocytes led to evolution of peripheral HIV. Particularly, we demonstrated the emergence of APOBEC-mediated mutations, which often drive early HIV evolution [48], suggesting that “astro-tropic” HIV strains could potentially emerge. One study found unique V3 region of the envelope gene in astrocytes compared to brain derived macrophages and multinucleated giant cells in patients who died of HIV-associated dementia [49] although an “astro-tropic” virus is speculative at this point and requires more research.

The tools to address the role of the brain as a reservoir for HIV have been limited. Studies in rhesus macaques have shown that even animals undergoing successful ART treatment have detectable SIV RNA in the CNS [50] and in pigtail macaques SIV CSF viral load was observed in an animal following latency reversing agents in the presence of ART [51,52]. There are also several rodent models for HIV and/or HIV proteins in the CNS. One such model injects NSG mice with human CD34+ cord blood hematopoietic stem cells which over time will differentiate into a human immune system. Following adult peripheral HIV infection this model assessed PBMCs migration in the context of HIV and are useful in the study of HIV treatments [53,54]. However, none of these existing models were appropriate to trace HIV from the brain to the periphery. Our model is a modification of that from the Goldman’s group [55] whereby immunocompromised *rag2*^*-/-*^ mice were injected with human glial progenitor cells (GPCs) on post-natal day 1 (PND1) mice. These cells proliferated and migrated throughout the lifespan of the mice to the extent that by one year of age the entire brain was engrafted with terminally differentiated human astrocytes and to a lesser extent oligodendrocytes [56,57]. Another group injected human fetal astrocytes into the striatum of adult nude mice to examine the potential of astrocytes for therapeutic purposes and found that these astrocytes survive, and similar to our adult model, do not migrate far from site of injection [58]. We modified these models to assess HIV in human astrocytes egressing from the brain to the periphery in either neonate or adult NSG mice following reconstitution with human PBMCs. This chimeric HuAstro/HuPBMCs NSG mouse model ensures primary viral infection in the CNS, allows a primary focus on astrocytes (as no other resident brain cell is humanized) and utilizes the use of full-length infectious virus. We recognize that the HuAstro/HuPBMCs model cannot address the contribution of infected myeloid cells (monocytes/macrophages/microglia), which play an important role in HIV neuroinvasion and HAND, to HIV spread outside of the brain [59–61]. It is highly likely that infected macrophages/microglia could release HIV into the CNS, along with astrocytes as shown here, which can then disseminate into peripheral organs further highlighting the important contribution of the CNS to the cure initiative.

Debate surrounds the role of astrocytes in HIV infection. While several studies have detected HIV in astrocytes from human post mortem brain and through in vitro infection [26,32,34,62,63], it has remained controversial whether HIV infects astrocytes and whether HIV DNA detected in astrocytes is replication competent. One study in particular using fixed and live-cell fusion assays of reporter virus showed no evidence for infection and suggested that signals of HIV in astrocytes many be due to astrocytes engulfing HIV infected macrophages [21]. This particular conclusion is based on using sensitive luciferase assay which may not detect signal from a very small fraction of cells and most critically the assay did not measure HIV integration (e.g. HIV entry through none- classical/non fusion pathway and escape from endosome) that is likely to occur in astrocyte infection of HIV. There is also no evidence to our knowledge of the ability of astrocytes to phagocytose macrophages in vivo. Most importantly in their reported model the astrocytes and the macrophages were not from the same donor, as such astrocytic phagocytosis of infected cells may be atypical of astrocyte behavior and a “non-physiologic system” is driving this reported phagocytosis of infected cells in vitro. Nonetheless, a number of groups including our findings here demonstrated HIV integration in astrocytes using Alu-PCR [31,32]. Further, HIV LTR-luciferase expression in astrocytes can be silent as a function of cell heterogeneity [64]. Here we have HIV infected astrocytes ex vivo with full length HIV virus (NL4-3 and IIIB) and transplanted them into the mouse brain. Given that astrocytes are trypsinized and washed prior to injection (removing membrane bound HIV) and injection of free virus (absence of astrocytes) did not lead to peripheral infection suggests that astrocytes are releasing infectious virus in vivo. Our animal model is complemented by our post-mortem human brain analyses of HIV+ patients under cART whereby: 1) Isolation of GFAP+ astrocytes via laser capture microdissection followed by amplification of integrated HIV DNA, 2) analyses of %GFAP+ astrocytes for HIV gag mRNA, and 3) analyses of %GFAP+ cells for HIV p24 all demonstrated presence of HIV in astrocytes of systemically aviremic patients. It is of interest to note that the reported rate of HIV infection in the hu-astro animal model and post-mortem tissue is comparable. A potential explanation is that our data demonstrates that the rate of astrocyte infection is very low and typically the majority of cells are not infected. Those that do become infected do so through HIV endocytosis and escape of HIV from the endocytic pathway to establish HIV integration, as demonstrated by others [24,29,30]. As such the rate limiting step is the rate of HIV escape from endosome to become integrated and that may be similar whether the infection is initiated in vitro or in vivo. Because astrocytes express robust β-catenin that inhibits HIV at the transcriptional level [36]. When signals inhibit β-catenin signaling, bursts of HIV occur. As such, we believe that there is a small pool of cells that harbor integrated HIV DNA and that pool is largely latent and once the opportunity is there a burst of HIV ensues. Therefore, both the animal approach and the human study reached the same conclusion that astrocytes can harbor HIV and the animal model further shows that this astrocyte-harbored HIV is capable of replication and spread.

HIV neuroinvasion in a natural course of infection occurs through peripheral virus entering the CNS. However, modeling this infection course experimentally would make it hard to distinguish egressed virus from the original peripheral infection. Our model allows the examination of brain derived HIV egress by initiating the infection in the CNS then assessing for HIV presence outside of the brain. Nonetheless, to demonstrate infection of astrocytes through the physiologic means of HIV neuroinvasion (periphery to CNS), we xenotransplanted uninfected astrocytes into the brain of NSG mice then reconstituted with HIV infected PBMCs. Under this experimental paradigm, we also find HIV infection of astrocytes. We also demonstrate that CD4+ T cells home into the CNS, become infected with astrocyte derived HIV then likely traffic out of the brain to support HIV egress into the periphery. It is likely that CD4+ T cells and not monocytes transport HIV to the periphery since monocytes differentiate into macrophages and migrate within a tissue but to date there is no evidence that macrophages would migrate between tissues (e.g. move from brain to lung/lymphoid organs and vice versa) although we do not exclude other immune cells contributing to HIV egress out of the brain. Though not addressed here, it would be interesting to determine whether astrocytes release free virus which can either infect infiltrating PBMCs, whether cells form a bridge to infect via cell-to-cell contact as has been shown going from PBMCs to astrocytes in vitro [22] or as of yet an unidentified method of infection.

Detection of HIV egress initiated from HIV infected astrocytes in the brain to peripheral organs under cART suggests that low level HIV production in the brain may contribute to the emergence of HIV viral blips, which are detected in the CSF and periphery during cART. cART was initiated prior to PBMC reconstitution and we used cART doses that are reported to support viral suppression [65], albeit cART in general has low penetrance into the CNS [66] and as such cART may not fully suppress HIV replication in the CNS. Although we did observe a dramatic reduction in HIV egress under cART, egress was not completely eliminated. This phenomenon is also observed in HIV+ patients in which about 20% of individuals on cART have detectable HIV in the cerebrospinal fluid (CSF) despite complete viral suppression in the periphery, including individuals on decade-long suppressive therapy [67,68]. This CSF viral escape is associated with neurological impairment, suggesting that residual HIV replication within the CNS under cART drives neuropathogenesis [69] and perhaps may also contribute to HIV genetic evolution within and outside of the brain.

Collectively, our studies demonstrate an in vivo role of brain astrocytes in supporting ongoing HIV replication, mediating HIV egress from CNS to peripheral organs through T cell trafficking in and out of the CNS, and potentially creating an environment within the CNS that is supportive for low level HIV production under cART. As astrocytes, microglia/ macrophages have all been shown to harbor productive HIV, the brain must be considered as an important reservoir/sanctuary site that under the appropriate signal(s) may become reactivated and spread HIV to peripheral organs, further complicating HV cure efforts.

## Materials and methods

### Ethics statement

All animal procedures were approved by Rush University Medical Center Institutional Animal Care and Use Committee (IACUC protocol 17–001) and conducted in accordance with the NIH guidelines for housing and care of laboratory animals. Rush University Medical Center is fully accredited by the AAALAC-International. Human peripheral blood mononuclear cells (PBMCs) were collected in accordance with institutional (original IRBL06080703, converted to 17111308-IRB02) and U.S. Government guidelines on human research. Human tissue sections were were collected as part of the IRB-approved for Rutgers University and the Manhattan HIV Brain Bank and National NeuroAIDS Tissue Consortium (NNTC).

### Astrocyte Cell Culture and HIV infection

Fetal-derived Normal Human Astrocytes (NHAs, Lonza, Walkersville, MD) were maintained in Astrocyte Growth Media (AGM) BulletKit (Lonza). Early passages (1–4) were used in these experiments. U138MG astrocytoma cell line was obtained from the American Type Culture Collection (ATCC, Manassas, VA), and propagated in Dulbecco's modified eagle's medium (DMEM; Thermo Fisher, Waltham, MA) supplemented with 10% HI-FBS serum (HI-FBS; Sigma, St. Louis, MO) and 1% penicillin-streptomycin (Thermo Fisher). Cells were maintained in a 5% CO_2_ humidified atmosphere at 37°C. Astrocytes were either primed with IFNγ (100ng/ml, R&D Systems, Minneapolis, MN) overnight then infected with HIV or HIV_VSV_ at 10ng/ml of p24 as indicated. Twenty-four hours post infection cells were washed and treated with new media. Seven days post infection; adherent cells were washed and removed by treatment with 0.24% (v/v) trypsin for 5 min, washed and suspended in sterile PBS for injection.

### Viral production

GFP-expressing HIV-1 (HIV) viral particles were produced by transfecting HEK 293T cells (ATCC) with 25μg of pNLENG1-IRES-70 (kindly provided by David N. Levy and constructed by John Kappes). This construct contains full length CXCR4-tropic HIV NL4-3 DNA with GFP expression under the control of IRES. VSVg pseudotyped HIV-1_NLENG1_ (HIV_VSV_) viral particles were produced by co-transfection of 25μg pNLENG1-IRES-70 and 5μg of pVSVg (Addgene, Cambridge, MA). Cells were transfected by the calcium-phosphate method. Briefly, 6-7h after transfection fresh DMEM supplemented with 10% HI-FBS and 1% penicillin/streptomycin. Forty eight and 72h post-transfection, the media was collected and centrifuged at 300 xg for 10min followed by filter through a 0.45-μm Millex-HV filter unit (Millipore, Billerica, MA). Media was then aliquoted and stored at -80ºC for long term storage. The CXCR4 tropic HIV-1 IIIB (NIH AIDS Reagent Program) was grown in 174xCEM cells (NIH AIDS Reagent Program). Media was collected, centrifuged at 300 xg for 10 min followed by filtering through 0.45- μm Millex-HV filter unit then aliquoted and stored in -80ºC for long term storage. HIV-1 p24 was quantified for each virus using immunofluorescent magnetic bead assay as described previously with slight modifications [70]. MagPlex carboxymethylated microspheres (Luminex Corporation, Madison, WI) were coupled with a high affinity anti-p24 monoclonal antibody (ImmunoDiagnostics, Inc., Woburn, MA) using Xmap antibody coupling kit (Luminex Corporation) as per instructions. Eight μl of the antibody coupled beads at 50 beads/μl concentration were incubated with 80μl of lysed (with 10% Triton X-100 (10%v/v) for 45 min at 37ºC) virus samples at 37ºC using a rotator at 30 rpm for 1h. Samples were washed twice with wash buffer by gently pipetting up and down and centrifuged at 8000 rpm for 2 min. Samples were incubated with RD-1 labeled anti-p24 KC57 antibody (1:200 dilution, Beckman Coulter, Indianapolis, MN) at room temperature and rotated for 1h at 30 rpm. Samples were again gently washed twice with wash buffer, suspended in assay buffer and run on an LSR II flow cytometer with BD FACSDiva software (BD Biosciences, San Jose, CA). Standard curve (p24 range, 0-30ng/ml) was prepared by using Alliance p24 standard (PerkinElmer, Waltham, MA). Analysis of flow cytometry data was done using FlowJo software (FlowJo LLC, Ashland, Oregon).

### Transplantation of astrocytes into mouse brain

NOD/SCID/IL-2rcγ^−/−^ and NOD.Cg-Prkdc^scid^IL2rγ^tm1Wjl^/SzJ (NSG) mice were purchased from The Jackson Laboratory (Farmington, CT) and housed under pathogen-free conditions. *Neonate injection*: newborn pups of either sex were injected within one day of birth with 200,000 HIV- or HIV_VSVg_+ HIV NHAs. Following cryoanesthesia, 4 injections of 50,000 cells in 0.5μl each were targeted to each of 4 locations in the forebrain subcortex as described previously [71]. Pups were returned to their mother and weaned at 5 weeks of age. *Adult injection*: adult males 5–6 weeks of age were anesthetized with ketamine/xylazine (90 mg/kg, 5 mg/kg, I.P.) and placed in a stereotaxic apparatus. Bilateral injections of astrocytes (50,000 cells/μl, pre-transplantation treatment as indicated) or free virus (1-2ng of p24/μl) were targeted to the striatum with coordinates relative to Bregma +0.5 A/P, +1.8 M/L, −3.0 D/V. One μl was injected into each hemisphere at 0.2 μl/min rate. The needle was left in place for one minute before injection and five minutes after and mice were given buprenorphine (0.1mg/kg) for pain relief following surgery. *Human PBMC reconstitution*: Research involving human subjects was conducted in accordance with institutional (IRBL06080703) and U.S. government guidelines on human research. PBMCs were isolated by density gradient centrifugation from HIV sero-negative human donor venous blood and injected IP with 2 × 10^7^ PBMCs. Monocytes were depleted using RosetteSep Human Monocyte Depletion Cocktail and CD4+ T cells by RosetteSep Human CD4+ Depletion Cocktail (STEMCELL Technologies, Cambridge, MA) and were injected IP with 2 × 10^7^ of the remaining PBMCs. Neonates were reconstituted at 6 weeks of age and adults were reconstituted 5 days after microinjection surgery. Four weeks following reconstitution, mice were anesthetized by CO_2_ inhalation then perfused by cardiac puncture with 30 ml ice-cold PBS for dissection of brain, lymph nodes, spleen and blood.

### Evaluation of Blood Brain Barrier Permeability

Evaluation of BBB permeability was performed as detailed [72]. Briefly, animals were placed under anesthesia and transcardially perfused with a 10mg/ml solution of FITC-Dextran (10kDA) (Sigma-Aldrich). Immediately after the perfusion was completed, the brains were removed, fixed and cryoprotected. The brains were segmented using a brain matrix and then embedded in OCT for cryosectioning. The segments containing the injection site were sectioned at 50 microns and visualized by a Nikon A1R scanning confocal microscope. Image analysis were performed using the Nikon NIS Elements Advanced Research imaging software. The detection of the fluorescence tracer in the CNS parenchyma was performed by using particle analysis and binary image conversion to exclude the vasculature. Pixel intensity in the parenchymal areas of the injection site were then measured as a function area. Thus, the results are presented as mm^2^ of tracer positive areas.

### PBMC Cell Culture and HIV Infection

PBMCs were isolated from HIV sero-negative human donor venous blood and isolated using SepMate PBMC Isolation (STEMCELL Technologies). Cells were cultured overnight with 20u/ml IL-2 (NIH AIDS Reagent Program) and 1 μg/mL soluble α-CD3 and α-CD28 antibodies (BD Biosciences) in Roswell Park Memorial Institute medium 1640 (RPMI; Thermo Fisher) supplemented with 10% HI-FBS serum (HI-FBS; Sigma) and 1% penicillin-streptomycin (Thermo Fisher). Cells were maintained in a 5% CO_2_ humidified atmosphere at 37°C. PBMCs were then infected with 2ng/1×10^6^ cells/ml of HIV-1-GFP. Cells were washed and maintained for one week in culture before reconstitution of 2 × 10^7^ cells per neonatal animal xenotransplanted with uninfected NHAs.

### Antiretroviral treatment

Animals were treated with a combination of three drugs previously shown to suppress viral loads [65]. Mice were given every other day IP injections of emtricitabine (FTC; Gilead; 211 mg/kg bodyweight), tenofovir disoproxil fumarate (TDF; NIH AIDS Reagent Program; 205 mg/kg bodyweight) and raltegravir (RAL; NIH AIDS Reagent Program; 56 mg/kg bodyweight) beginning 3 days prior to reconstitution and continuing every other day for 4–5 and a half weeks as indicated.

### Viral outgrowth assay

Spleens were collected from mice at the time of perfusion. Spleens were mashed and red blood cells were lysed using Red Cell Lysis Buffer (Sigma, St. Louis, MO) to obtain a single-cell suspension of splenocytes. Splenocytes were stimulated with either Phytohaemagglutinin *(*PHA, Sigma, 4 μg/ml) or with 1 μg/mL soluble α-CD3 and α-CD28 antibodies (BD Biosciences) and suspended in complete RPMI 1640 media (Lonza) supplemented with 10% FBS (Gemini Bio Products, West Sacramento, CA), 1% penicillin/streptomycin (Sigma), and 20 U/mL IL-2 (NIH AIDS Reagent Program). Cultures were maintained for 14 days. To determine if harvested splenocytes released replication competent virus supernatant was collected through centrifugation at 1500 rpm for 5 min, further centrifuged at 5000 rpm for 5 min to clear off cell debris and incubated with new PBMCs from same donor used for mouse reconstitution. These cells were stimulated with α-CD3 and α-CD28 in complete RPMI overnight prior to splenocyte supernatant addition and maintained for 7 days.

### Real-time PCR

Animals were perfused as described above. Following dissection, DNA and RNA was isolated from striatum of one brain hemisphere, spleen, cervical lymph nodes, peripheral lymph nodes and PBMCs isolated from blood by DNeasy Blood and Tissue Kit (Qiagen, Hilden, Germany) and Trizol (Thermo Fisher) respectively per manufactures instructions. Nucleic acids were quantified using nanodrop2000 (Thermo Fisher). RNA was further treated with DNaseI (Sigma) for 15 min at RT to remove DNA contamination, and subsequently, DNaseI was inactivated by heating at 70°C for 15 min. cDNA was synthesized from 0.5–2 μg of RNA using Qscript supermix (Quanta Biosciences, Gaithersburg, MD). Real-time PCR reactions were performed in a 20μl solution containing 10 μl TaqMan Gene Expression Master Mix (Life Technologies, Carlsbad, CA), 900-nm primers and 500-nm probe, and 2 μl cDNA or 250-500ng of genomic DNA samples. Reactions were performed in an Applied Biosystems 7900HT sequence detection system (Thermo Fisher Scientific) using SDS2.3 software, under the following reaction conditions: 50°C for 2 min, 95°C for 10 min, followed by 45 cycles of 95°C for 15 s and 60°C for 1 min. Samples were run in duplicates, and standards were included wherever necessary. To generate standard curve for HIV DNA copy number, a 500bp gBlock gene fragment encompassing the gag region was synthesized and to generate standard curve for HIV RNA copy number, a 337bp gBlock gene fragment of env region was synthesized (Integrated DNA Technologies, Coralville, IA). The fragment was suspended in sterile TE buffer at dilutions ranging from 50 to 1 × 10^6^copies/μl. Copy number was calculated using the DNA copy number Web tool (Thermo Fisher) and converted to RNA copy number by multiplying by 2. The dilutions were aliquoted and stored at −80°C. The primers and probe were designed using the PrimerQuest tool (Integrated DNA Technologies). The sequence of the primers was as follows: GAG-F, 5′-CCCAGAAGTGATACCCATGTT; GAG-R, 5′-GCTTCCTCATTGATGGTCTCT and GAG-probe, 5′/56-FAM/ATTTGCATG/ZEN/GCTGCTTGATGTCCC/3IABkFQ; ENV-F, 5’-GGAGGAGGAGATATGAGGGATAA; ENV-R, 5’-TCTGCACCACTCTTCTCTTTG and ENV-probe, 5′/56-FAM/ACCATTAGG/ZEN/AGTAGCACCCACCAA/3IABkFQ; human specific huGAPDH-F, 5’-GGTGTGAACCATGAGAAGTATGA; huGAPDH-R, 5’-GAGTCCTTCCACGATACCAAAG and huGAPDH-probe, 5′/56-FAM/AGATCATCA/ZEN/GCAATGCCTCCTGCA/3IABkFQ; mouse specific msGAPDH-F, 5’-AACAGCAACTCCCACTCTTC; msGAPDH-R, 5’-CCTGTTGCTGTAGCCGTATT and msGAPDH-probe, 5’/56-FAM/TTGTCATTG/ZEN/AGAGCAATGCCAGCC/3IABkFQ; human specific huGFAP-F, 5’-ACCCAGCAACTCCAACTAAC; huGFAP-R, 5’TTCTCTCCTTCCTCCTCATTCT and huGFAP-probe, 5’/56-FAM/CATGGCCAG/ZEN/CAGCTTGCGTT/3IABkFQ; mouse specific msGFAP-F, 5’AACAACCTGGCTGCGTATAG; msGFAP-R, 5’TCTCGAACTTCCTCCTCATAGAT and msGFAP-probe, 5’-/56-FAM/TGGCTCGTG/ZEN/TGGATTTGGAGAGAA/3IABkFQ; hu cytochromeB mtDNA-F, 5’TAGCAATAATCCCCATCCTCCATATAT; hu cytochromeB mtDNA-R, 5’ACTTGTCCAATGATGGTAAAAGG. Real time PCR products were run on 1% agarose gel stained with ethidium bromide and captured using a gel documentation system. For sequencing, a 527bp GAG fragment was amplified from gDNA using AmpliTaq Gold Master Mix (Applied Biosystems, Foster City, CA) and primers GAG-F-CCTTCAGACAGGATCAGAAGAAC and GAG-R-CTCCTACTGGGATAGGTGGATTA. Reaction conditions were, 95°C for 10 min, followed by 35 cycles of 95°C for 15 s and 60°C for 1 min. Samples were run through Quiquick PCR purification kit (Qiagen) and sequenced.

### Immunolabeling

Mouse brains were fixed in 4% paraformaldehyde for 24 hours at 4 ºC then kept in 30% sucrose solution at 4 ºC. Forty μm thick coronal sections were used in these studies. Tissue sections were incubated in primary antibodies overnight treatment at room temperature. Primary antibodies are as follows: Anti-huGFAP (1:200–500, BioLegend, San Diego, CA, 837202), anti-GFAP (1:1000, Abcam, Cambridge, UK, ab4674), anti-huNuclei (1:200, Millipore, MAB1281), anti-human CD3 (1:200, Agilent, Santa Clara, CA, A0452) and anti-p24 (Abcam, ab53841). After washing sections of primary antibody they were incubated in secondary antibody (1:200, Life Technologies) for 1 h at room temperature. After washing the sections were covered with ProLong Gold Antifade Mountant with DAPI (Thermo Fisher).

### Mouse RNAscope and DNAscope

Brain tissues were prepared and assayed according to protocols provided by Advanced Cell Diagnositics. Briefly, mouse brains were perfused then placed in increasing concentrations of sucrose up to 30% before embedding in OCT embedding media. Tissue was sectioned at 10 μm and immediately mounted onto slides, which were then baked at 60C in a hybridization oven and then fixed to slides using 4% paraformaldehyde. Tissue pretreatment included incubation with hydrogen peroxide, boiling in target retrieval buffer, and protease treatment. Sections were then incubated for two hours with probes targeting HIV RNA, HIV DNA, and/or human-specific GFAP (ACD Bio, Inc). Signal was developed using a Multiplex Fluorescent Detection Kit v2 (ACD Bio, Inc.) according to manufacturer’s protocol. Sections that were stained for immunofluorescence were washed after signal development and stained according to immune labeling described above.

### Flow cytometric analysis

Single-cell suspensions of splenocytes were stained with LIVE/DEAD Fixable Violet Dead Cell Stain (Life Technologies) according to the manufacturer’s instructions. Cells were washed with PBS and stained with CD3-APC-H7, CD4-APC, CD8-PERCP-Cy5.5, CD16-Alexa Fluor 700, CD14-PacBlue and HLA-ABC-PECy7 (BD Biosciences) for extracellular stains. For intracellular detection of HIV-1 core Ag p24 cells were fixed and permeabilized using BD Cytofix/Cytoperm Solution Kit (BD Biosciences) according to the manufacturer’s instructions before staining with 1 μl per sample of HIV-1 core Ag–RD-1 (Beckman Coulter). When cell number was limiting, a minimum of 5 × 10^3^ live gated events were collected in each sample and analyzed to maintain cell number between samples for comparison of different T cell populations. Data were collected on an LSR II flow cytometer with BD FACSDiva software (BD Biosciences) and analyzed using FlowJo Software (TreeStar, Ashland, OR).

### Single genome amplification and sequencing

To assess viral evolution, we performed single genome amplification (SGA) and sequencing of HIV *gag* from three adult animals that were xenotransplanted with astrocytes infected for 5 days ex-vivo with HIV NLENG1-IRES. DNA was extracted from spleen collected 28 days post-xenotransplantation and subjected to serial dilutions until single viral genomes were amplified, as previously described [43,44]. A 473 bp region of *gag* was amplified by nested PCR using first round forward primer 5’-TAT AGT ATG GGC AAG CAG GG-3’, reverse primer 5’-CTC CTA CTG GGA TAG GTG GAT TA-3’, and second round forward primer 5’-CCT YCA GAC AGG ATC AGA AC-3’, and reverse primer 5’-GTA GTT CCT GCT ATG TCA CTT CC-3’. The PCR mixture contained 2 ul 10X High Fidelity Buffer, 0.8 ul 50mM MgSO_4_, 0.4 ul 10 mM dNTPs, 0.2 μl 20 μM forward and reverse primer, and 0.1 ul Platinum Taq DNA Polymerase High Fidelity (Invitrogen). First round PCR conditions were 94°C for 2 min, followed by 35 cycles of 94°C for 15 sec, 55°C for 30 sec, and 68°C for 1 min and a final extension of 10 min at 68°C. Second round PCR conditions were identical except for 45 cycles and a 57°C annealing temperature. PCR products were visualized on a gel and positive wells were directly sequenced. Sequence chromatograms were inspected using Sequencher software for mixed bases; sequences that contained ambiguous bases were excluded from further analysis. Gag nucleotide sequences were aligned using Geneious software. Highlighter plots, APOBEC signature mutations, Poisson distribution, star-like phylogeny, and most recent common ancestor (MRCA) were determined using the online LANL HIV database tools Highlighter and Poisson-Fitter (https://www.hiv.lanl.gov/content/sequence/HIGHLIGHT/highlighter_top.html; https://www.hiv.lanl.gov/content/sequence/POISSON_FITTER/poisson_fitter.html).

### Human tissue sections

Sections of 25 μm thickness were processed for immunofluorescence and confocal microscopy as described below (n = 8, four uninfected and four HIV-infected with no viral replication detected for years, 5–23 years). 12 sections were analyzed (1.5±0.75 x 0.91±0.66 cm average size analyzed) per subject. Peripheral and CSF viral load was measured using COBAS Taqman (Roche, Basel, Switzerland) per manufacturer’s instructions. Limit of detection was 20 copies/ml/.

### In situ GAG RNA analysis, DNA, p24 and GFAP staining

Brain tissue sections obtained from uninfected and HIV infected individuals were subjected to staining for DAPI, Alu-repeats, GFAP, HIV-RNA, and HIV-p24 protein. Briefly, ALU (Cy5 CGG TCC CAA AGT GCT CGG ATT ACA) and NEF (Biotin—GCA GCT TCC TCA TTG ATG G) DNA probes were obtained from PNA Bio (Newbury Park, CA). 10nm for each probe was diluted in TBS and incubated at 55ºC for 2 hours. Slides were then washed in preheated 55°C TBC for 30 minutes. 26 FITC (Thermo Fisher, streptravidin, 434311) was then added directly to the slides for 30 minutes. Then, RNAscope kit from Advance Cell Diagnostics (Newark, CA) was used according to the manufacture’s protocol. Specifically RNAscope Probe—HIV-gagpol (317691), RNAscope 2.5 HD Detection Reagents-RED (322360), RNAscope Wash Buffer Reagents (310091), RNAscope H202 & Protease Plus Reagents (322330), RNAscope Target Retrieval Reagents (322000) were used. Once RNA staining was finished, slides were placed in 100% ethanol for 30 seconds and then air dried for 5 minutes. Slides were washed 3 times in PBS. Samples were then blocked for 30 minutes in blocking solution. GFAP (Cell Signaling, Danvers, MA, 36070S) and HIV P24 (GenetTex, Inc., Irvine, CA, GTX40774) was then added at 1:500 dilution overnight. Samples were then washed 3 times in PBS. Secondary antibodies were diluted 1:1000 in PBS (Streptavidin 700 S21383 and 350 Mouse A11045). Samples were then washed three times in PBS and mounted with Prolong Diamond Antifade Mountain medium with DAPI (Thermo Fisher, P36962). To detect HIV integrated DNA, several conditions are necessary: first, co-localization of HIV-Nef DNA with DAPI and Alu repeat probes was essential. Second, lack of co-localization with GFAP, HIV-p24 and minimal co-localization with HIV mRNA was required. The analysis was performed using NIS element program (Nikon, Japan).

### Laser capture microdissection

Tissue sections were stained for DAPI, GFAP, and HIV-RNA as described above. Nuclei with positive cytoplasmic RNA were isolated using laser capture microdissection using a Leica 6500 system (Germany). Between 200 to 500 nuclei were isolated to run the subsequent Alu-gag PCR to quantify the numbers of integrated copies. As negative control astrocytes from uninfected individuals were used. As controls, OM-10, HL60, and PBMCs cells were used as well as standard curve diluting DNA in Hela cell DNA.

### Alu-gag PCR

Integration of HIV into the host genome was detected by Alu-gag PCR as described previously with minor variations [73]. The system was calibrated using OM-10 cells and diluted OM-10 cells into millions of uninfected Hela cells to quantify the lowest numbers of copies possible in 10^6^ to 10^9^ cells.

### Statistical analysis

Statistical analysis was performed with consultation of Ethan Ritz from the Rush biostatical core. All data are presented as mean ± SEM. All statistical analysis was performed using GraphPad Prism Software 5.00 using Kruskal-Wallis nonparametric test with Dunns *post hoc* or two-tailed Mann Whitney-U test. *P* values < 0.05 were considered significant.

## Supporting information

S1 Fig Amplification plots of HIV DNA and RNA from organs isolated from neonate mice post-NHA xenotransplantation. (**a-e**) Real-time PCR analysis of DNA extracted from the organs indicated from neonates for HIV DNA and human GAPDH transcript. (**f-i**) Real-time PCR analysis of RNA extracted from organs indicated from neonates for HIV and human GAPDH transcript. PCR products were run on gel and are shown in **Fig** 3. Insets are real-time PCR analysis for human GAPDH for the corresponding plot.
(TIF)

S2 Fig Amplification plots of HIV DNA and RNA from organs isolated from adult mice post-NHA xenotransplantation. (**a-f**) Real-time PCR analysis and PCR products run on gel of HIV DNA from the brain and peripheral sites as indicated in adult animals injected with HIV- or HIV_VSVg_+ NHAs. (**h-j**) Real-time PCR analysis and PCR products run on gel of HIV RNA from the brain and peripheral sites as indicated in adult animals injected with HIV- or HIV_VSVg_+ NHAs. PC indicates Positive Control for primers. Insets are real-time PCR analysis for human GAPDH for the corresponding plot. PCR products were run on gel and are shown in **Fig** 4.
(TIF)

S3 Fig Amplification plots of HIV DNA and RNA from organs isolated from adult mice post-NHA xenotransplantation. (**a-d**) Real-time PCR analysis from DNA or RNA and from organ as indicated for adult mice xenotransplanted with HIV- or HIV+ NHAs. (**e-h**) Real-time PCR analysis from DNA or RNA and from organ as indicated for adult mice xenotransplanted with HIV- or HIV_VSVg_+ U138 astrocytoma cell line. (**j-l**) Real-time PCR analysis from DNA or RNA and from organ as indicated for adult mice xenotransplanted with HIV- or HIV_IIIB_+ NHAs. (**m-p**) Real-time PCR analysis from DNA or RNA and from organ as indicated for adult mice injected with HIV- or HIV_VSVg_+ free virus. PC indicates Positive Control for primers. Insets are real-time PCR analysis for human GAPDH for the corresponding plot. PCR products were run on gel and are shown in **Fig** 5.
(TIF)

S4 Fig Peripheral HIV infection infects astrocytes in the neonatal xenotransplantation model. Additional images from different neonatal mice injected with uninfected NHAs and reconstituted with HIV+ huPBMCs and sacrificed 4 weeks later immunostained for human astrocytes (huGFAP; red), HIV p24 (green) and Nuclei (DAPI, blue). Arrows indicate co-localization of huGFAP and p24. *n* = 6. Scale bar, 20μm.
(TIF)

S5 Fig cART treatment blocks astrocyte infection in the neonate xenotransplantation model. Neonatal mice were injected with uninfected NHAs. cART treatment began 1 day prior to reconstitution and continued every other day for 4 weeks till sacrifice. Animals were reconstituted with HIV+ huPBMCs. (**a**) RNAscope for huGFAP (red), HIV (green) and DAPI (blue). (**b**) Immunoflurescence staining for huGFAP (red), p24 (green) and DAPI (blue). *n* = 3 animals, 4 and 6 coronal sections were analyzed per animal for RNAscope and immunofluorescence respectively. Scale bar, 50μm.
(TIF)
