# Supplementary figures and images for: HIV infects astrocytes in vivo and egresses from the brain to the periphery
Source: PLoS Pathog. 2020 Jun 11;16(6):e1008381. doi: 10.1371/journal.ppat.1008381 (PMC7289344; doi:10.1371/journal.ppat.1008381)

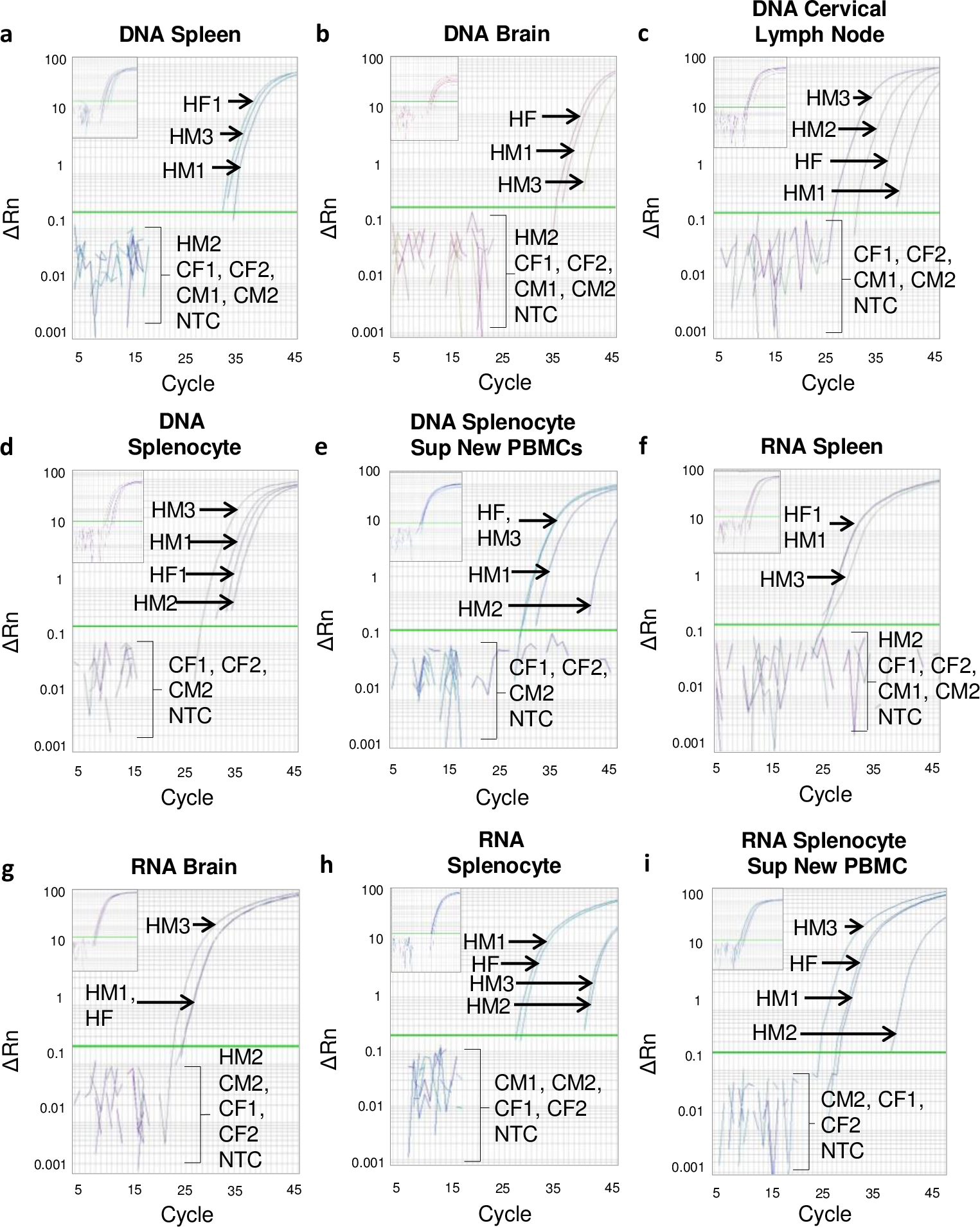

Supplement: S1 Fig — (a-e) Real-time PCR analysis of DNA extracted from the organs indicated from neonates for HIV DNA and human GAPDH transcript. (f-i) Real-time PCR analysis of RNA extracted from organs indicated from neonates for HIV and human GAPDH transcript. PCR products were run on gel and are shown in Fig 3. Insets are real-time PCR analysis for human GAPDH for the corresponding plot. (TIF) [file ppat.1008381.s001.tif]

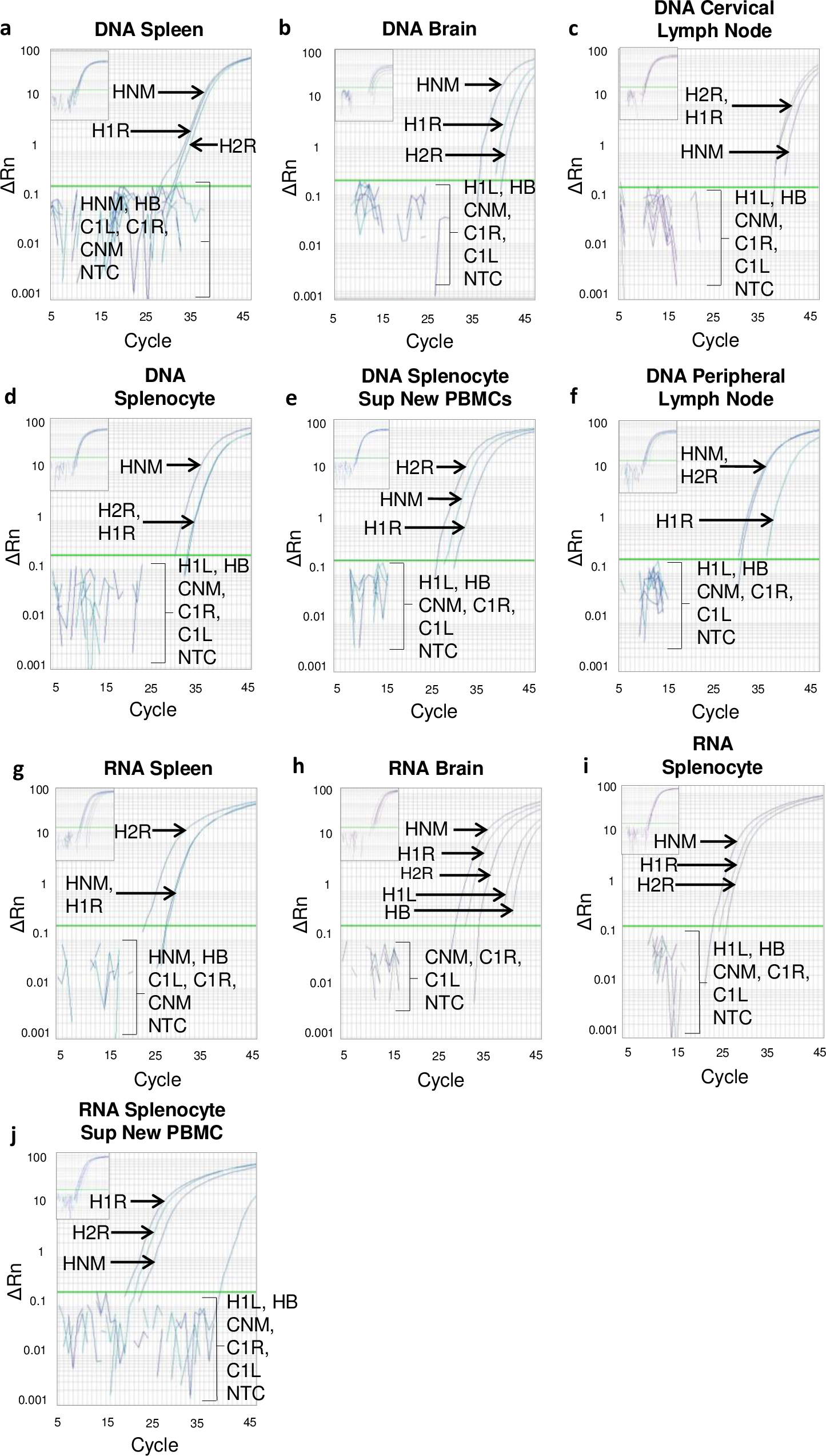

Supplement: S2 Fig — (a-f) Real-time PCR analysis and PCR products run on gel of HIV DNA from the brain and peripheral sites as indicated in adult animals injected with HIV- or HIVVSVg+ NHAs. (h-j) Real-time PCR analysis and PCR products run on gel of HIV RNA from the brain and peripheral sites as indicated in adult animals injected with HIV- or HIVVSVg+ NHAs. PC indicates Positive Control for primers. Insets are real-time PCR analysis for human GAPDH for the corresponding plot. PCR products were run on gel and are shown in Fig 4. (TIF) [file ppat.1008381.s002.tif]

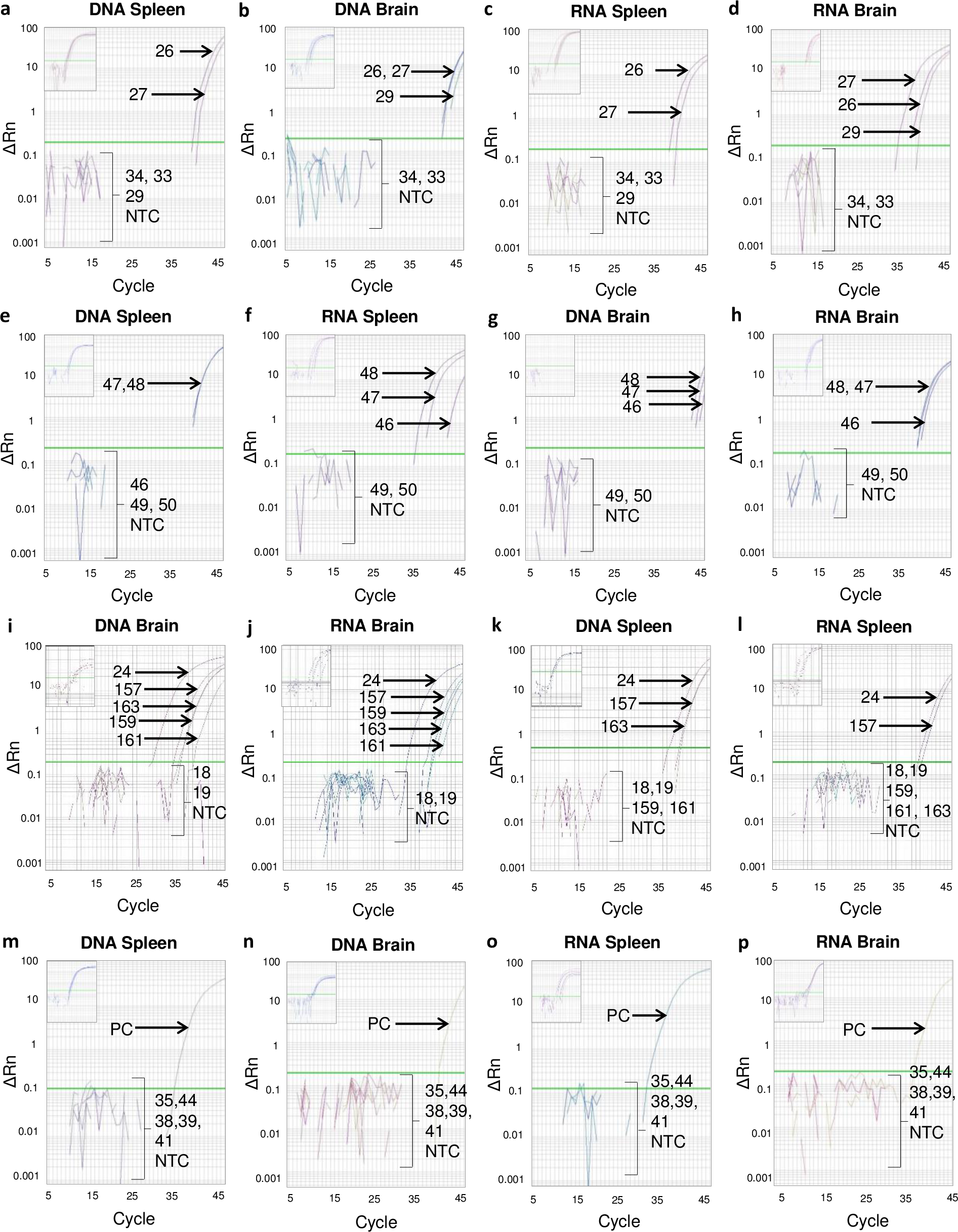

Supplement: S3 Fig — (a-d) Real-time PCR analysis from DNA or RNA and from organ as indicated for adult mice xenotransplanted with HIV- or HIV+ NHAs. (e-h) Real-time PCR analysis from DNA or RNA and from organ as indicated for adult mice xenotransplanted with HIV- or HIVVSVg+ U138 astrocytoma cell line. (j-l) Real-time PCR analysis from DNA or RNA and from organ as indicated for adult mice xenotransplanted with HIV- or HIVIIIB+ NHAs. (m-p) Real-time PCR analysis from DNA or RNA and from organ as indicated for adult mice injected with HIV- or HIVVSVg+ free virus. PC indicates Positive Control for primers. Insets are real-time PCR analysis for human GAPDH for the corresponding plot. PCR products were run on gel and are shown in Fig 5. (TIF) [file ppat.1008381.s003.tif]

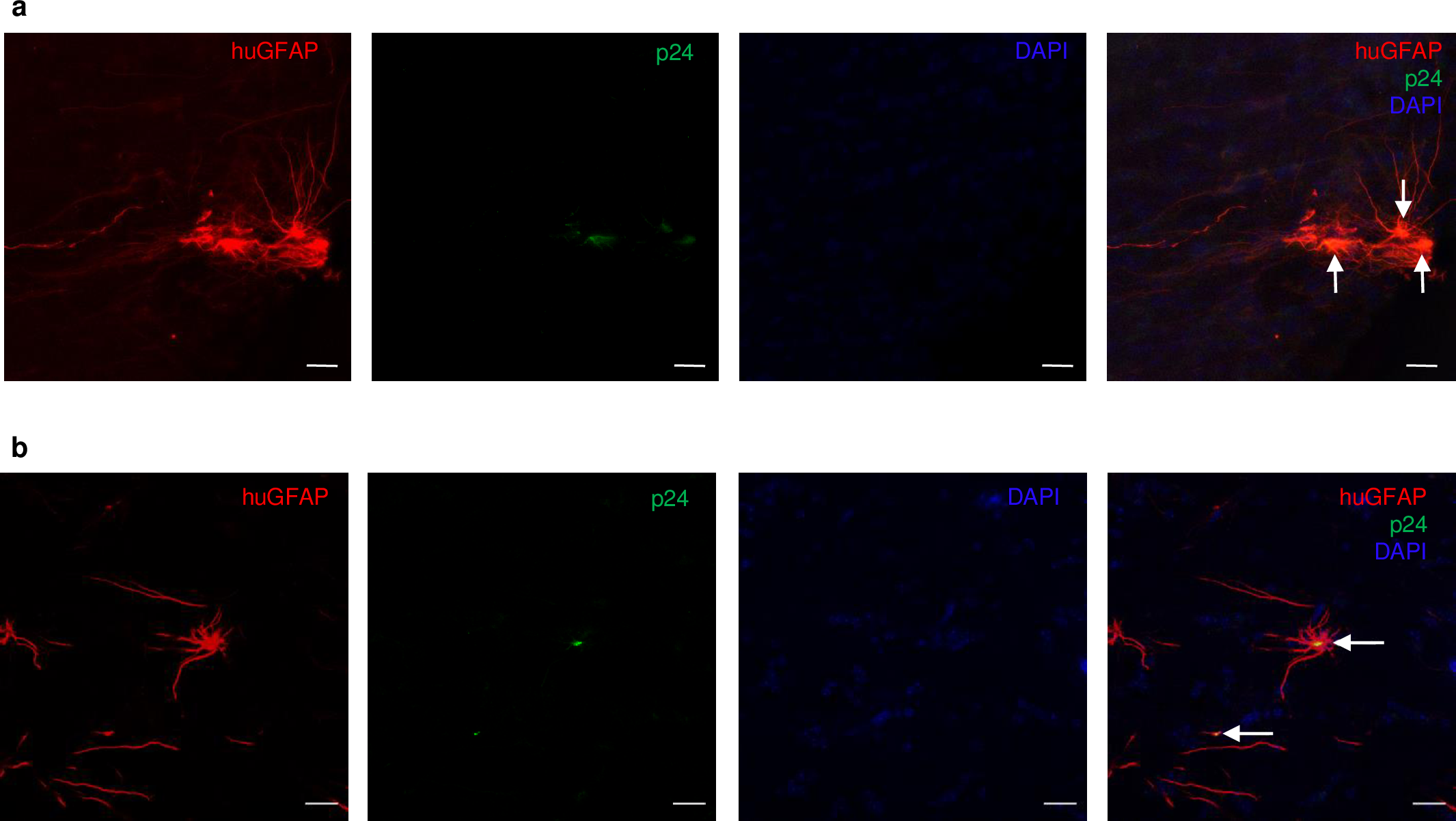

Supplement: S4 Fig — Additional images from different neonatal mice injected with uninfected NHAs and reconstituted with HIV+ huPBMCs and sacrificed 4 weeks later immunostained for human astrocytes (huGFAP; red), HIV p24 (green) and Nuclei (DAPI, blue). Arrows indicate co-localization of huGFAP and p24. n = 6. Scale bar, 20μm. (TIF) [file ppat.1008381.s004.tif]

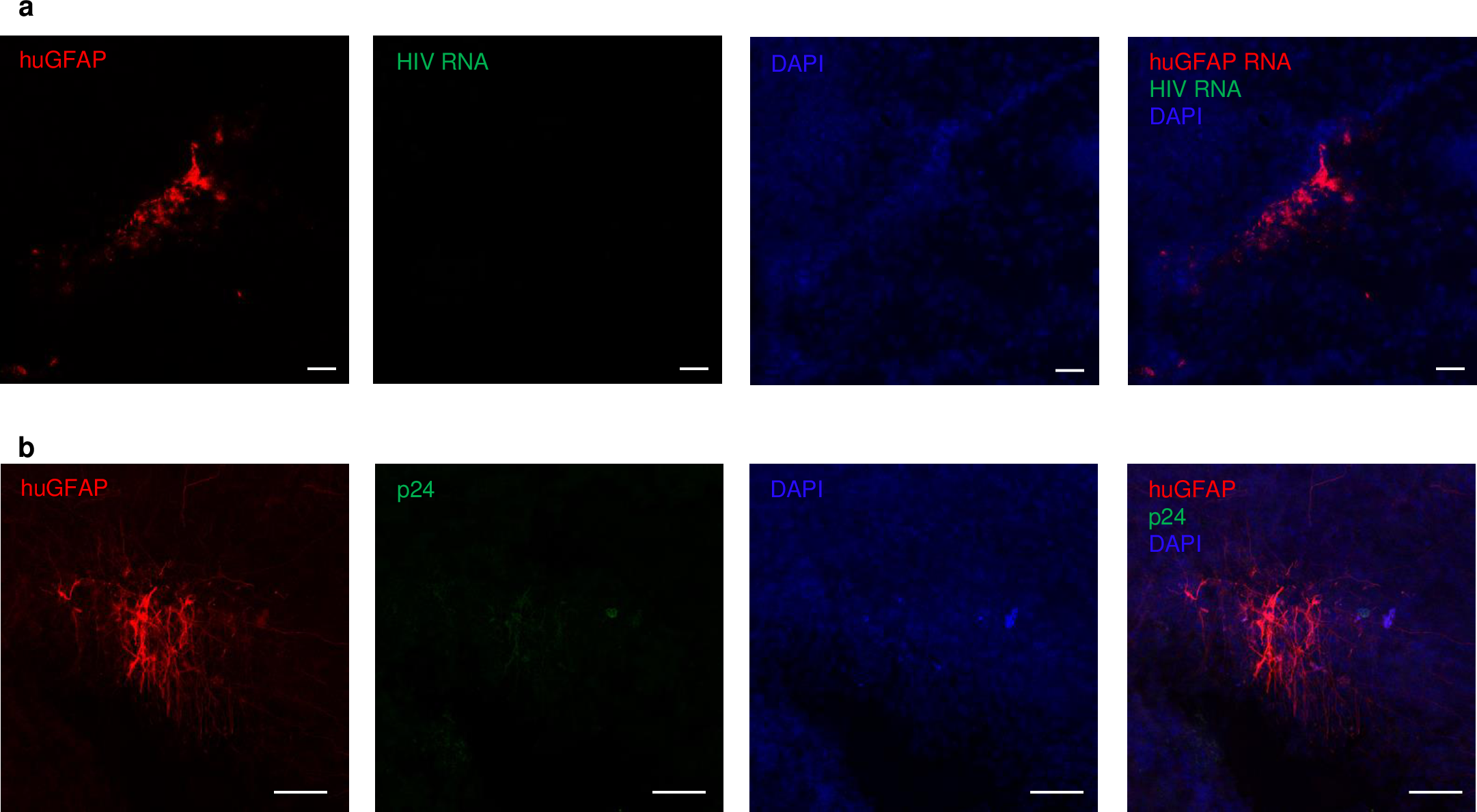

Supplement: S5 Fig — Neonatal mice were injected with uninfected NHAs. cART treatment began 1 day prior to reconstitution and continued every other day for 4 weeks till sacrifice. Animals were reconstituted with HIV+ huPBMCs. (a) RNAscope for huGFAP (red), HIV (green) and DAPI (blue). (b) Immunoflurescence staining for huGFAP (red), p24 (green) and DAPI (blue). n = 3 animals, 4 and 6 coronal sections were analyzed per animal for RNAscope and immunofluorescence respectively. Scale bar, 50μm. (TIF) [file ppat.1008381.s005.tif]
